# A novel immunocompetent transgenic mouse model of DHF reveals Syk-mediated Th2-polarized cytokine storm as a key driver of vascular leakage

**DOI:** 10.1080/22221751.2025.2531178

**Published:** 2025-07-07

**Authors:** YuYa Wang, Wei Yang, YuanSong Yang, HuiHui Cheng, Rui Xiong, Xi Wu, Nan Xu, ShuNan Liu, Zhe Qu, Chen Ling, Yuan Cao, Yanwei Yang, HaoYang Zhao, WenDa Gu, Yong Wu, SuSu Liu, Yining Wang, Yu Wang, SanLong Wang, Rui Fu, WeiJin Huang, Hongning Zhou, Wei Wei, Jing An, YouChun Wang, ChangFa Fan

**Affiliations:** a Institute for Laboratory Animal Resources, National Institutes for Food and Drug Control (NIFDC), Beijing, People’s Republic of China; b Basic School for Medical Science, Capital Medical University, Beijing, People’s Republic of China; c Novogene Co., Ltd., Beijing, People’s Republic of China; d Department of Microbiology & Infectious Disease Center, School of Basic Medical Sciences, Peking University Health Science Center, Beijing, People’s Republic of China; e Division of HIV/AIDS and Sexually Transmitted Virus Vaccines, National Institutes for Food and Drug Control (NIFDC), Beijing, People’s Republic of China; f Institute of Virology and AIDS Research, First Hospital, Jilin University, Changchun, People’s Republic of China; g National Center for Safety Evaluation of Drugs, National Institutes for Food and Drug Control (NIFDC), Beijing, People’s Republic of China; h College of Life Science school, Northwest university; Provincial Key Laboratory of Biotechnology of Shaanxi Province, Xi’an, People’s Republic of China; i Yunan Institute of Parasitic Diseases, Yunan, People’s Republic of China; j Institute of Medical Biology, Chinese Academy of Medical Sciences and Peking Union Medical College, Kunming, People’s Republic of China

**Keywords:** Dengue virus, Htim4 receptor, dengue hemorrhagic fever, hemorrhage, cytokine storm, th2-bias

## Abstract

Dengue virus (DENV) infection remains a critical global health threat, with DENV-2 being the most virulent serotype capable of causing lethal dengue hemorrhagic fever (DHF), a severe complication characterized by plasma leakage and hemorrhagic manifestations. While the search for viral receptors and immunocompetent animal models has persisted since the first recorded outbreak in 1779, significant gaps remain. Here, we establish the first immunocompetent murine model of DHF with intact innate/adaptive immunity by generating hTim4-transgenic C57BL/6J mice. This model recapitulates fatal DHF complications seen in humans, including systemic hemorrhage, dengue encephalitis and intestinal ischemia/gangrene. Integrated single-cell RNA sequencing and spatial transcriptomics analysis of hemorrhagic gut lesions demonstrated that DENV-2 infection induces Syk protein overexpression, leading to enhanced Th2 cytokine secretion and impaired hemostatic regulation. This cascade enhances vascular permeability, promotes plasma leakage, and drives multiorgan hemorrhage, a mechanism corroborated by parallel analyses of human DHF tissues. Critically, Th2-biased cytokine storm mirrors clinical findings in severe human dengue cases. Our work not only identifies hTim4 as a functional DENV-2 receptor but also provides a mechanistically grounded platform for DHF pathogenesis studies, bridging critical gaps between preclinical models and human immunopathology.

## Introduction

Approximately half of the global population is at risk of dengue virus (DENV) infection, posing a significant worldwide public health concern [1–4] that surpasses HIV as one of the top 10 health threats for 2019 according to WHO (https://www.who.int/health-topics/dengue-and-severe-dengue#tab=tab_1). The dengue epidemic is on the rise due to factors such as global warming and unplanned urbanization since dengue outbreak in 1779 [5]. Particularly in 2023, there have been over 5 million reported infections globally, including 5000 deaths, nearing a historic peak. New regions across the world, including southern Europe, are being affected [6]. Notably, in the updated pathogens that could spark the next pandemic issued by WHO in August 2024, dengue virus was back on the list [7].

DENV infection can lead to classical dengue fever (DF), severe dengue hemorrhagic fever (DHF), and even dengue shock syndrome (DSS), characterized by high fever, intense joint and muscle pain (hence DHF's nickname “broken bone fever”), systemic hemorrhagic tendency, thrombocytopenia, capillary leakage [8,9] and neurological complications [10]. The main challenges in combating the dengue epidemic include an unclear understanding of its pathogenesis and antibody-dependent enhancement (ADE), along with a lack of specific treatment options and ineffective vaccines for prevention [11]. Currently only two dengue vaccines have received approval; one being Dengvaxia vaccine which has been associated with severe adverse events following vaccination [12–14] and the other being Qdenga vaccine which was licensed in Indonesia in August 2022 before later gaining approval in Europe [15–17]. However, the long-term data regarding their preventive efficacy and safety remains necessary. For decades, there has been a persistent need to establish an ideal animal model for dengue virus [18], particularly a murine model. Despite significant progress made in using various animal species to develop DENV infectious models, certain limitations remain inherent to these models [18–23]. Rhesus macaques and tree shrews exhibit low viremia levels, while bonnet macaques, marmosets, chimpanzees, and Yucatan miniature pigs fail to display overt clinical manifestations. Adult wild-type mice are resistant to DENV infection and hard to simulate hallmark symptoms of DHF or DSS, even at high viral doses. The AG129 remains the exclusive murine platform selective recapitulating hemorrhagic pathology, however, its congenital immunodeficiency leads to dysregulated immune responses, rendering it unsuitable for investigating DENV immune pathogenesis and restricting its application to the evaluation of vaccine-induced neutralizing antibodies, which necessitates passive transfer of antibodies from immunized donors [18–23]. This has resulted in a critical bottleneck in hemorrhagic fever preventive and therapeutic development.

Notably, host receptors serve as the molecular portals for viral invasion, and the functional validation of these receptors is a key entry point for constructing susceptible animal models and elucidating infection mechanisms. Prior studies have identified numerous candidate molecules for DENV receptors [20]. However, these candidates have no functional validation evidence *in vivo*, lacking genetically modified murine models harboring these receptors [20,24], which substantially impedes the credibility of these molecules as dengue virus receptors and their applications in pathogenic mechanisms, the development of vaccines and antiviral drugs. Through a systematic literature review and analysis, it was demonstrated that within the Tim receptor family, only Tim1 and Tim4 mediate DENV invasion *via* the phosphatidylserine-dependent phagocytosis pathway [24]. Candidate receptors DC-SIGN serves as a critical mediator in antigen presentation and the initiation of immune activation cascades [21]. In contrast, Tim4 may contribute to disease progression by modulating antiviral immunity through immune responses and viral internalization [24]. Therefore, Tim1 and Tim4 receptor were selected to develop genetically modified murine models. Animal infection experiments confirmed that Tim1 transgenic mice were not susceptible to DENV, whereas the Tim4 receptor molecule significantly enhanced host susceptibility. Consequently, this study successfully established a stable and heritable hTim4 overexpression immunocompetent mouse model, achieving for the precise simulation of DENV infection pathology in a mammalian system. This model presents three breakthrough features: (1) It shows higher susceptibility to DENV-2 (50 PFU) infection than wild-type adult mice [18,20], with viral loads comparable to those in the plasma of human severe patients; (2) It systematically reproduces the core pathological phenotypes of DHF/DSS, including multi-organ hemorrhage (with fatal hemorrhage in the digestive tract as a typical manifestation), disseminated intravascular coagulation (DIC), and dengue encephalitis-like injury; (3) It detects a Th2-type cytokine storm (the phenomenon impaired in AG129 mice) highly consistent with that in human severe cases [25–28], providing a quantifiable window for the study of immunopathological mechanisms. These findings not only provide direct evidence for the receptor function of Tim4 through *in vivo* validation but more importantly establish an animal research platform capable of parallel analysis of the entire chain of DENV infection-immunity-pathology.

## Results

### Generate and validate immunocompetent htim4-transgenic murine models

**Figure 1.**
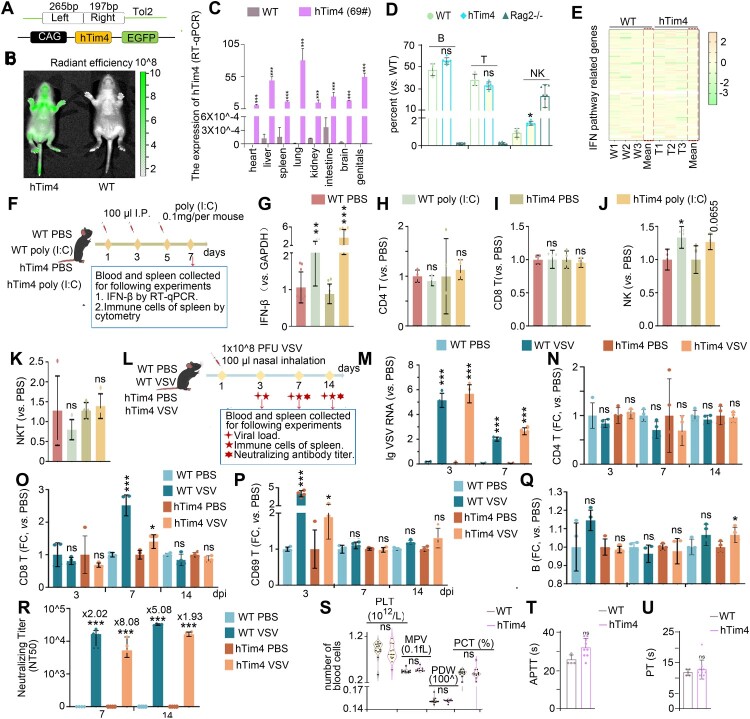
hTim4 transgenic mouse model with intact immune and coagulation systems. (A) the strategy used to generate hTim4 transgenic mice (hTim4 mice). Zygotes from C57BL/6J mice (WT) were injected with hTim4 Tol2 constructs. (B, C) verification of hTim4 expression. EGFP fluorescence confirmed systemic expression of hTim4 in newborn wild-type WT (gray) mice (n = 3) and hTim4 (green) mice (n = 3), visualized by radiant efficiency on IVIS Spectrum CT Imaging System (B). The RNA of heart, liver, spleen, lung, kidney, intestine, brain and reproductive organs collected from 6-week-old WT (n = 3) and hTim4 mice (n = 3) were quantified by RT-qPCR (C). (D) the proportions of immune cells. The proportions of B cells, T cells and NK cells by flow cytometry between the hTim4 group (n = 4) and WT group (n = 6), with Rag2^-/-^ group (n = 8) serving as positive control. (E) genes expression. No differential expression was observed among genes related to the IFN pathway. (F-K) the innate immune reactions simulated by poly(I:C). Experimental design (F). 100 μl of poly(I:C) were injected into each mouse on days 1, 3, and 5 between the poly(I:C) WT (n = 4) and poly(I:C) hTim4 (n = 4) mice. Equal volumes PBS were done as control. The blood and spleen were collected for further studies. IFN-β level by RT-qPCR on day 7. (G). The CD4 T cells (CD4+CD8-), CD8 T cells (CD4-CD8+), NK cells (CD3-CD49+) and NKT cells (CD3+CD49+) in the spleen tissue were counted by flow cytometry on day 7 (H-K). (L-R) the adaptive immune reactions infected with VSV. Experimental design (L). 100 μl VSV (1×10^8^ PFU) were nasal inhabited into each mouse (VSV WT, n = 12; VSV hTim4, n = 12). Equal volumes PBS were done as control. The blood and spleen were then collected for following assays. Blood viremia (M). The CD4 T cells (CD4+CD8-), CD8 T cells (CD4-CD8+), CD69 cells (CD69+) and B cells (CD3-CD19+) in the spleen tissue were counted by flow cytometry on 3, 7, 14 dpi (N-Q). The neutralizing antibody titer by NT50 (R). (S-U) the intact coagulation function in hTim4 mice. No significant differences were found in PLT, PDW, PCT (H), APTT (I), and PT (J). data: mean ± SD. Statistical analysis: C, D, G-K, M-U: Two-sided unpaired Student's *t*-test. *P<0.05; **P<0.01; ***P<0.001. The exact *P* values were numerically documented in Table S4

It has been reported that hTim4 may be a potential receptor of DENV virus [20,24]. To address the lack of immunocompetent dengue-susceptible murine models, a linearized plasmid containing the cDNA of hTim4 gene and EGFP was employed to inject C57BL/6 embryos and three founders 17#, 31#, and 69#, were obtained, (Figure 1A and Table S1). Only founder 69# exhibited stable germline transmission and high hTim4/EGFP expression (Figure 1B,C). To establish a dengue-susceptible model that accurately recapitulates clinical manifestations while preserving intact immune and coagulation functions, we rigourously evaluated the immune competence and hemostatic profiles of hTim4-transgenic C57BL/6J mice (termed as hTim4 mice). In the immune quiescent state, hTim4 mice exhibited immune cell levels similar to those of WT mice, while much higher than that of the Rag2^-/-^ deficient mice (positive controls) (Figure 1D and Figure S1A). RNA-seq analysis revealed minimal transcriptional alterations (185 upregulated and 179 downregulated genes out of a total of 29,253 genes, <1.2% affected) with no significant disruption of the IFN pathway, as determined by Reactome function enrichment analysis comparing wild-type C57BL/6J mice (WT mice) with hTim4 mice (Figure 1E and Figure S1B,C). In Immunologically activated state, the dsRNA analogue poly(I:C) significantly induced IFN-β and CD69+ NK (CD3-CD49+) cells increase [29,30] in both WT (*P*<0.01) and hTim4 (*P*<0.001) mice (Figure 1F-J), while no significant changes were observed in CD4 T (CD4+CD8-), CD8 T (CD4-CD8+) and NKT (CD3+CD49+) cells (Figure 1H-K); VSV infection has been demonstrated to effectively evaluate adaptive immunity [31,32] (Figure 1L). Viral dynamics showed peak viremia at 3 dpi and clearance by 7 dpi in WT and hTim4 mice infected with VSV (Figure 1M and Table S2). Significant upregulation of CD8 T cells (7 dpi) and CD69 activation marker (3 dpi) was observed (Figure 1O, P). hTim4 mice exhibited increased B cell counts at 14 dpi (*P*<0.05 vs WT, *P* = 0.119) (Figure 1Q) and higher VSV-neutralizing titers [33] (NT50: 1/8,080-1/19,300) compared to WT (NT50:1/20,200-1/50,800) (Figure 1R). Collectively, comprehensive immune profiling demonstrated preserved immune competence. Coagulation parameters, including platelet (PLT) counts, activated partial thromboplastin time (APTT), prothrombin time (PT), MPV, PDW, and PCT confirmed intact hemostasis in hTim4 mice (Figure 1S-U and Figure S1D, E). In summary, this novel hTim4 transgenic mouse was immunocompetent amd enables compromising immune or coagulation functions that may be suitable for dengue research.

### Htim4 mediates DENV-2 infectivity via receptor function in transgenic mice

**Figure 2.**
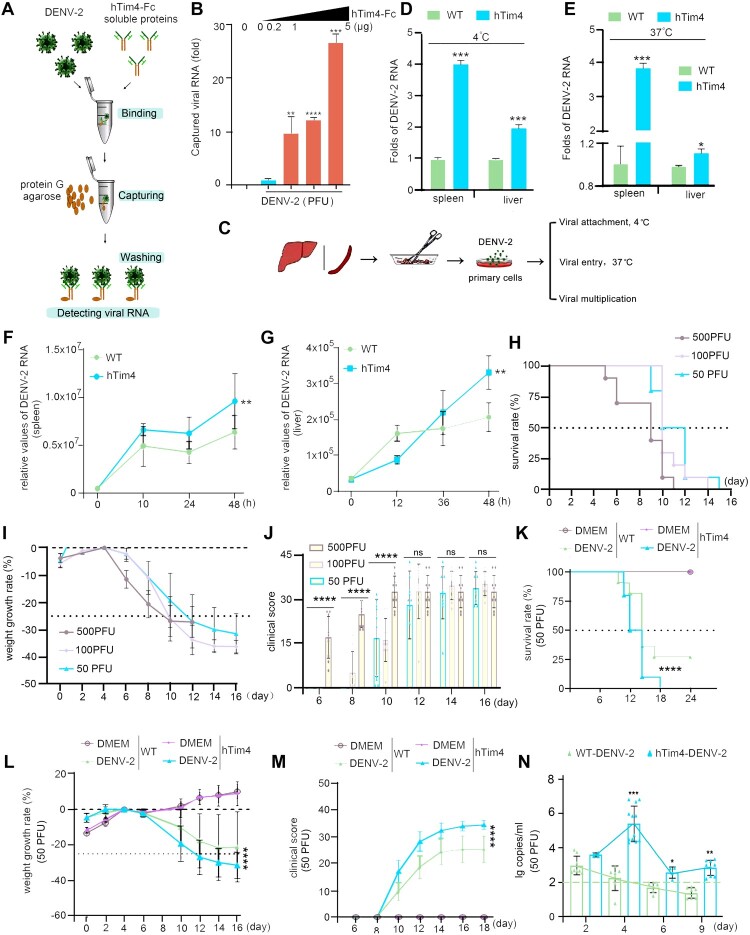
hTim4 mediates DENV-2 infectivity through its receptor function in hTim4-transgenic mice. (A) The procedures for *in vitro* capturing assays. (B) *In vitro* assays. the relationship between hTim4-Fc content and the amount of virus captured. DENV-2 (500 PFU) was mixed with 0, 0.2, 1 and 5 (μg) hTim4-Fc soluble proteins to form the DENV-2/hTim4 complex which was then isolated using protein G agarose beads. The extracted viral RNA was used to detect virus capture efficiency. (C-E) *ex vivo* assays. Primary spleen and liver cells were infected by DENV-2 via hTim4 molecular interaction. Primary cells were inoculated for 48 h (10^5^ cells), followed by infection with DENV-2 (500 PFU) for attachment assay at both 4°C and 37°C for 2 h each. (F, G) Viral proliferation*.* Viral proliferation in primary spleen or liver cells infected with DENV-2 over time periods ranging from 0 to 48 h post-infection respectively. (H-J) Dose optimization (500, 100, 50 PFU) for DENV-2 I.C. infection. Survival curves at different infection dose. (H). Weight growth rates. Weight loss exceeding 25% was considered fatal (I). Clinical scores (J). (K-N) hTim4 mice are highly susceptible to dengue virus infection. Both WT and hTim4 mice were challenged with 50 PFU (50 PFU, I.C.) of DENV-2 Tr.1751. An equal volume of DMEM was inoculated as the control. All infected mice died within 18 dpi. (K). Weight growth rates decreased since 6 dpi. (>25% weight loss is considered fatal (L)). Clinical scores of these hTim4 mice were higher than those of WT mice (M). Blood viremia levels peaked at 4 dpi., lasted beyond 9 dpi. (N). Data: mean ± SD. Statistical analysis: B, D, E, K, N: Two-sided unpaired Student's *t*-test. F, G, I, L, M: Two-Way ANOVA. H, K: Mantel-Cox. *P<0.05; **P<0.01; ***P<0.001. The exact *P* values were numerically documented in Table S4.

To elucidate the function of hTim4, a series of assays were carried out. The dose-dependent binding of hTim4-Fc protein to DENV-2 (Tr1751 strain) (Figure 2A, B) indicates its direct involvement in viral recognition, corroborating previous findings [24]. Further validation at the cellular level revealed that primary spleen and liver cells from hTim4 mice (Figure 2C) exhibited a 4-fold increase in viral attachment efficiency at 4℃ (Figure 2D), a 2-fold increase in viral entry (Figure 2E), and significantly enhanced viral replication (Figure 2F, G), confirming that hTim4 drives the infection cascade by promoting viral adsorption, endocytosis, and replication [24]. To validate these findings *in vivo*, we established an infectious model. And to circumvent the limitations of high-dose infection protocols (e.g. conventional AG129 models) that may mask authentic pathogenesis, this study attempted to develop low-dose infection research system. Following preliminary optimization, subcutaneous and intraperitoneal inoculation routes demonstrated suboptimal efficacy, thus the intracranial (I.C.) challenge strategy could produce expected results. We identified 6-week-old hTim4 mice as highly susceptible to 50 PFU DENV-2, after systematically screening 3–11-week-old hTim4 mice and testing intracranial (I.C.) inoculation doses (50–500 PFU) (Figure S2), manifesting rapid lethality (50% mortality at 12 dpi and 100% at 18 dpi, Figure 2H), severe weight loss (>25% by day 6, Figure 2I), and progressive clinical deterioration (significantly elevated symptom scores from 8 dpi, Figure 2J). Crucially, this model recapitulated key features of severe human dengue, notably sustained viremia: viral RNA was detected in blood from 2 to 9 dpi, peaking at 10^4.5^–10^7^ copies/mL on day 4, with 25% of individuals reaching 10^6^ copies/mL (Figure 2K), dynamically aligning with clinical severe cases [34]. In summary, this study not only supports hTim4 as a critical functional receptor for DENV-2 infection but also successfully develops an intracranial infection animal model with immunocompetence.

### DENV-2 intracranial infection in htim4 mice simulates dengue-like encephalitis

**Figure 3.**
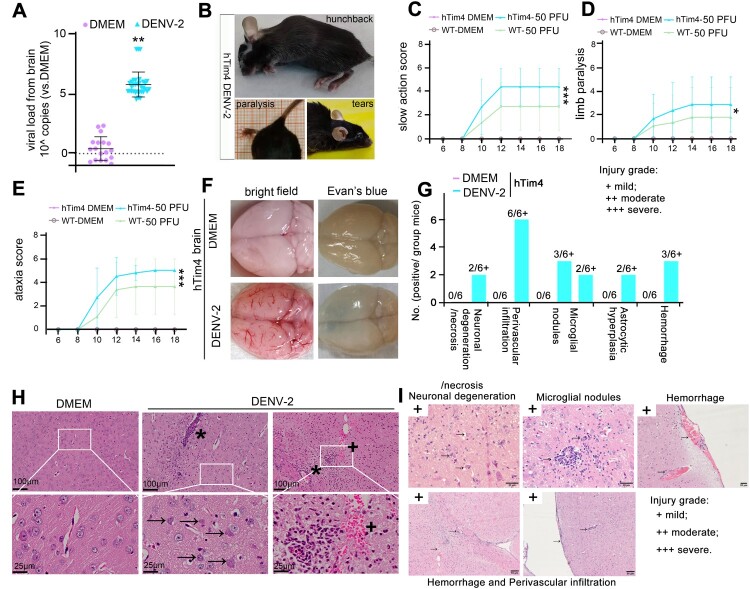
The hTim4-transgenic murine model of DENV-2 intracerebral challenge recapitulates key clinical features of dengue-associated encephalitis. Six-week-old hTim4 mice were intracranially inoculated with DENV-2 at a dose of 50 PFU (DENV-2 group, n = 44), while the control group received DMEM infection (DMEM group, n = 22). (A-E) Visible symptoms and clinical score. Visible symptoms in hTim4 mice infected with DENV-2 included viral load of brain (A), hunchback, tearing (B), slow action (C), limb paralysis (D) and ataxia (E). (F) Brain hemorrhage was observed through naked eye observation and Evan's blue staining. (G) The injury grade among 6 randomly selected DENV-2 infected mice subjected to pathological examination. (H,I) Pathological features in brain with DENV-2 infected mice. (H) perivascular infiltration (*), neuronal necrosis (→), and bleeding (+). (E) Pathological features of lung hemorrhage. bleeding (+), pulmonary hemosiderosis (▴), and edema (★). (I) Neuronal degeneration/necrosis (+), perivascular infiltration (+), microglial nodules (+), astrocytic hyperplasia (+) and hemorrhage (+) were graded mild. (I) Data are presented as mean ± SD. Statistical analysis: A, Two-sided unpaired Student's *t*-test. C-E, Two-way ANOVA. *P<0.05; **P<0.01; ***P<0.001. The exact *P* values were numerically documented in Table S4.

Clinically, some patients have been observed to develop dengue encephalitis. Therefore, we aim to investigate whether hTim4 mice exhibit similar symptoms. We observed that DENV-2 infected mice exhibited clinical signs, including hunching, emaciation, tearing (Figure 3A,B), and characteristic neurological symptoms such as slow action (Figure 3C and Table S6) and limb paralysis accompanied by ataxia (Figure 3D,E and Table S6), phenotypes closely resembling the “breakbone fever” manifestations of dengue hemorrhagic fever [8,9]. Pathological evaluation of brain tissues from six infected hTim4 mice demonstrated the following lesions: neuronal degeneration or necrosis (mild, +) in two cases, perivascular infiltration cell infiltration (mild, +) in six cases, microglial nodule formation (mild, +) in three cases, astrocytic hyperplasia (mild, +) in two cases, and hemorrhage (mild, +) in three instances (Figure 3F-I). Hemorrhagic features were confirmed through triple-validation methods, including bright-field microscopy, Evans blue permeability assay, and H&E staining (Figure 3F-I), with lesions localized to cortical regions (+), sporadic neuronal necrosis (→), and perivascular infiltration (*). These patterns precisely mirrored the spontaneous parenchymal microhemorrhage profile of human dengue encephalitis [10]. Despite pathological involvement in multiple brain regions (e.g. thalamus and basal ganglia) [10] the observed injury severity (mild) suggested that direct DENV-2-induced brain damage was not the primary cause of mortality. In conclusion, the hTim4 mouse model faithfully recapitulates the neuroinvasive phenotypes and encephalitis-specific pathological progression of intracranial DENV-2 infection, providing a robust disease model for investigating dengue neuropathogenesis. The lethal outcome in this model may stem from systemic pathological cascades triggered by viremia rather than direct central nervous system injury.

### DENV-2 intracranial infection in htim4 mice recapitulates DHF manifestations

**Figure 4.**
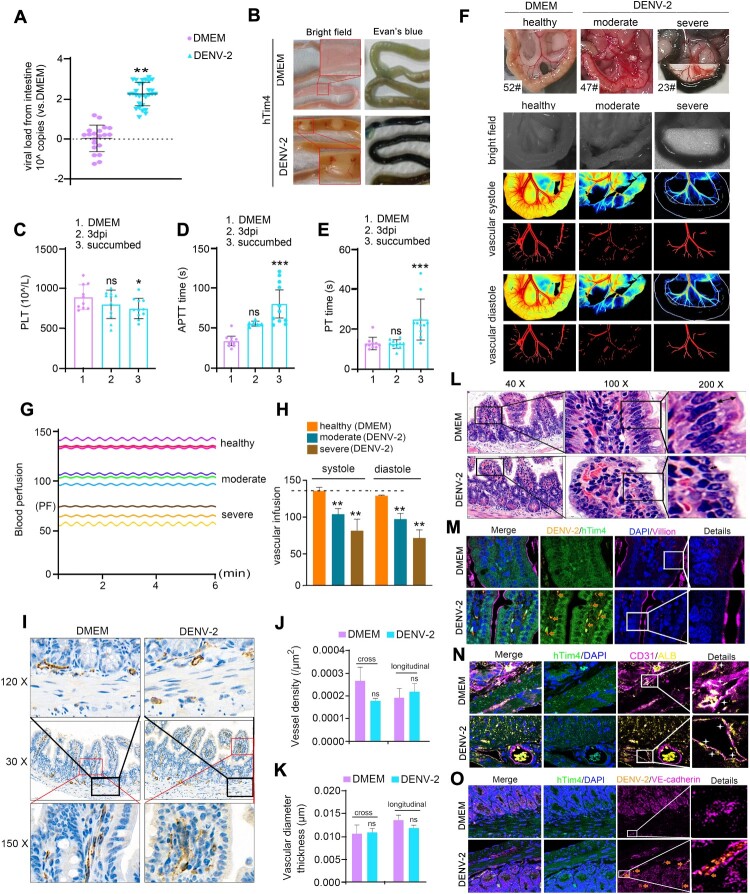
The hTim4-transgenic murine model of DENV-2 intracerebral challenge recapitulates pathophysiological hallmarks of DHF/DSS. (A) Viral load of intestine. (B) Intestine hemorrhage in hTim4 mice with DENV-2 infection. (C-E) Blood coagulation function characteristics after DHF induced by viral infection showed PLT decrease (C), APTT (D) and PT (E) prolongation. (F-H) Blood flow features and blood perfusion pressure were assessed using HR-LSCI laser speckle blood flow imaging system. In the DMEM group, all mice had healthy intestines (F). In the DENV-2 group, hTim4 mice infected with DHF were divided into moderate and severe groups based on disease severity. The color signal indicated decreased blood flow in the moderate group and stasis in the severe group; a stronger red signal did not indicate an increased number of blood vessels but rather faster blood flow velocity (F). Blood perfusion pressure was measured during systole and diastole stages (G, H). (I-K) No significant pathophysiological changes were observed in blood vessels between the DENV-2 infected group and DMEM group. Immunohistochemical staining with CD31 was used to label the intestinal vessels (I). The vascular density (J) and diameter (K) of both cross and longitudinal sections of the intestine were measured. (L) Pathological features of intestinal bleeding (3DHISTECH P250 FLASH system). Intestinal villi injury, crypt collapse, disorderly arrangement of intestinal columnar epithelial cells, and cytoplasm atrophy (↔) were observed in the DENV-2 group. (M-O) Molecular characteristics related to changes in vascular permeability were investigated using Zeiss LSM900 with Airyscan2 system. Nuclear disarrangement (blue), hTim4 (green), DENV-2 (brown, ➫) cytoplasm atrophy and intestinal villi injury (purple, M); albumin (yellow) leakage from vessels (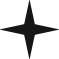, N); VE-cadherin (purple, ➫) expression remained unchanged (O). Data are presented as mean ± SD. Statistical analysis: A, C-E, H: Two-sided unpaired Student's *t*-test. J, K: Two-sided unpaired Student's *t*-test (welch).*P<0.05; **P<0.01; ***P<0.001. The exact *P* values were numerically documented in Table S4.

Systemic hemorrhage, as a core clinical manifestation of DHF, frequently progressed to disseminated intravascular coagulation (DIC), also named DSS [8]. However, recapitulating this complex pathological cascade in animal models has proven challenging [20,21]. Our study demonstrates that DENV-2-infected hTim4 transgenic mice precisely mimic these clinical features, with characteristic hemorrhagic foci observed in multiple organs including brain (Figure 3), intestine, lung, heart, liver, and spleen (Figure 4A, B and Figure S3A-C). Systematic coagulation analysis revealed prolonged clotting time from 4 days post-infection (dpi), significantly increased tail bleeding points by 8 dpi (*P* < 0.05, FigureS3D,E), accompanied by thrombocytopenia (Figure 4C), reduced mean corpuscular volume (MCV) (*P* < 0.05, FigureS3F) and over 50% prolongation of APTT and PT (Figure 4D,E). The observed APTT prolongation (>1.5× reference range) and PT extension (> 3s) closely mirror diagnostic criteria for human DIC, validating the clinical relevance of this model. Notably, concurrent reductions in WBC and NEU counts (Figure S3G,H) suggest immune dysregulation may exacerbate coagulation disorders. To further elucidate the hemorrhage dynamic changes, taken intestinal hemorrhage for example, we divided the DENV-2 mice into mild, moderate and severe state/condition based on pathological intestinal scoring (Table S7) and intestinal perfusion pressure drop rate (Table S3). Using high resolution laser speckle contrast imaging (HR-LSCI) [35], we quantitatively characterized DSS hemodynamics: intestinal blood flow velocity progressively declined (Table S3, Figure 4F, S3I) with disease severity, accompanied by 28.48% (moderate) and 42.23% (severe) reductions in perfusion pressure respectively (Figure 4G,H) – consistent with clinical DSS staging criteria (early phase: 10–20% decline; late phase: >30% decline) [36–38]. Immunohistochemical staining verified that platelet endothelial cell adhesion molecule-1 (PECAM-1/CD31) labelling showed no significant differences in intestinal microvascular density or structural integrity between infected and control groups (Figure 4I-K), suggesting preserved vascular architecture. HE staining analysis revealed severe disruption of intestinal villi architecture in critical cases, manifested by blood cell extravasation, nuclear atypia, and cytoplasmic atrophy (Figure 4L). Immunofluorescence staining assays discovered DENV-2 particles (brown, ➫) co-localizing with host receptor hTim4 (green) at lesion sites in infected group, directly demonstrating that those pathological changes are caused by DENV-2 infection (Figure 4M,N): villous layer shrinkage (purple) and epithelial disorganization (irregular nuclear arrangement, blue, Figure 4M), albumin (ALB, yellow) penetrates beyond the vessel wall (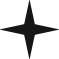, CD31, purple) (Figure 4N). Intriguingly, VE-cadherin (purple, ➫) expression showed no statistical difference between groups (Figure 4O), implying a VE-cadherin independent mechanism underlying vascular leakage. In essence, we found that hTim4 mice recapitulates faithfully the clinic-pathological progression of coagulation dysfunction, microcirculatory failure, and multi-organ hemorrhage induced by DENV-2 infection, demonstrating that hTim4 mice might serve as a pivotal experimental model for investigating the hemorrhagic pathogenesis of DHF/DSS.

### Single-cell and spatial omics uncover syk-driven immune-mediated hemorrhage

**Figure 5.**
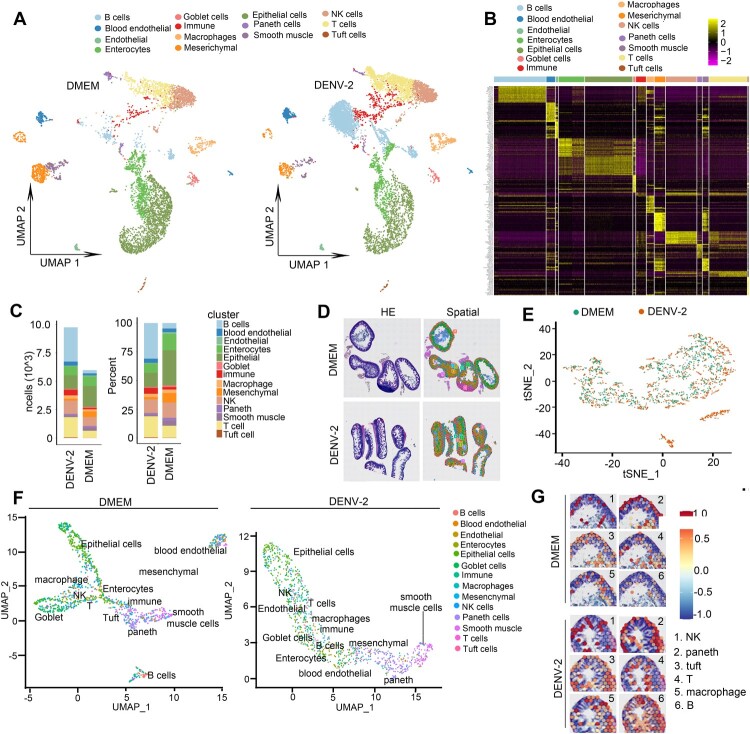
Molecular signatures of intestinal bleeding in mice with DHF. (A-C) Molecular signatures of scRNA-seq analysis. We utilized the Cell Ranger software's (V6.0) cellranger count module for alignment, filtering, barcode counting, and UMI counting to generate feature barcode matrices and identify clusters. Furthermore, cells with fewer than 200 genes or lower than 99 UMI, as well as those with mitochondrial gene proportions exceeding 25%, are filtered using Seurat (v4.0.0). Following the standardization and normalization of sample information, the samples were categorized into 14 distinct cell types via UMAP mapping visualization. UMAP revealed the differences in expression and distribution patterns among cell types between the DMEM and DENV-2 groups (A). The heatmap of the top 20 genes for each cell type (B). The cell count and percentage of different cell types were compared between the DMEM and DENV-2 groups (C). (D-G) Molecular signatures of spatial sequencing analysis. Each 10 μm frozen intestine tissue section from the DMEM group and DENV-2 group was placed on one visium gene expression slide capture area per slide. Bright-field images were acquired following the Spatial Transcriptomics procedure, and sequencing libraries were constructed using the visium Spatial Gene Expression Slide & Reagent kit (10 × Genomics) for Novaseq PE150 sequencing. The expressions of sequencing libraries underwent normalization, dimensionality reduction, spot clustering, and differential expression analysis using Seurat software (D). tSNE mapping visualized the expression and distribution of all gene spots across the samples (E). UMAP mapping demonstrated the expression and distribution differences between DMEM and DENV-2 groups (F). Cell types’ spatial location distribution. NK cells, paneth cells, tuft cells, T cells, macrophages, and B cells being located together showed the evidence of immune response presence (G).

Currently, the core regulatory genes mediating intestinal ischemia and hemorrhagic phenotypes, along with their spatial distribution characteristics, remain unclear. To systematically investigate the molecular association between intestinal gene expression profiles and vascular permeability abnormalities, this study integrated single-cell RNA sequencing (scRNA-seq) and spatial transcriptomics – the latter enabling in situ capture of spatial localization information for gene expression within tissue microenvironments to establish molecular mapping. Experiments involved parallel analysis of uninfected (DMEM control) and DENV-2-infected mouse intestinal tissue samples. Raw sequencing data underwent standardized processing, dimensionality reduction (UMAP/tSNE), cell clustering, and differential expression gene (DEG) screening through the Seurat pipeline. The scRNA-seq successfully identified 14 intestinal cell subtypes: B cells, hemogenic endothelial cells, endothelial cells, enterocytes, epithelial cells, goblet cells, immune cells, macrophages, mesenchymal cells, NK cells, Paneth cells, smooth muscle cells, T cells, and Tuft cells (Figure 5A). Classification accuracy was validated by subtype-specific molecular markers (top 20 DEGs, log_2_|FC|≥0.25) (Figure 5B). Quantitative analysis revealed significant immune cell expansion in DENV-2-infected group versus control: absolute counts of B cells (3022 *vs* 281), T cells (1786 *vs* 617), NK cells (1170 *vs* 794), macrophages (283 *vs* 181), and unclassified immune cells (502 *vs* 128) were markedly elevated (Figure 5C). Notably, however, relative proportions of macrophages (2.9% *vs* 3.03%) and NK cells (12.01% *vs* 13.3%) showed slight decreases in infected group (Figure 5C), suggesting dynamic equilibrium regulation among immune subsets. To further validate these findings, spatial transcriptomics data were integrated to construct intestinal tissue molecular maps. tSNE visualization demonstrated significant spatial distribution heterogeneity between DENV-2 and DMEM control groups across 14 cell subtypes (Figure 5D-F). Key immune cells (NK, T, B, macrophages, and cluster cells) exhibited pronounced spatial co-localization in infected group (Figure 5G and Figure S4A-N), aligning with scRNA-seq identified immune expansion. This finding is consistent with clinical observations in patients, demonstrating an increase in immune cell count without substantial immune cell aggregation and infiltration.

**Figure 6.**
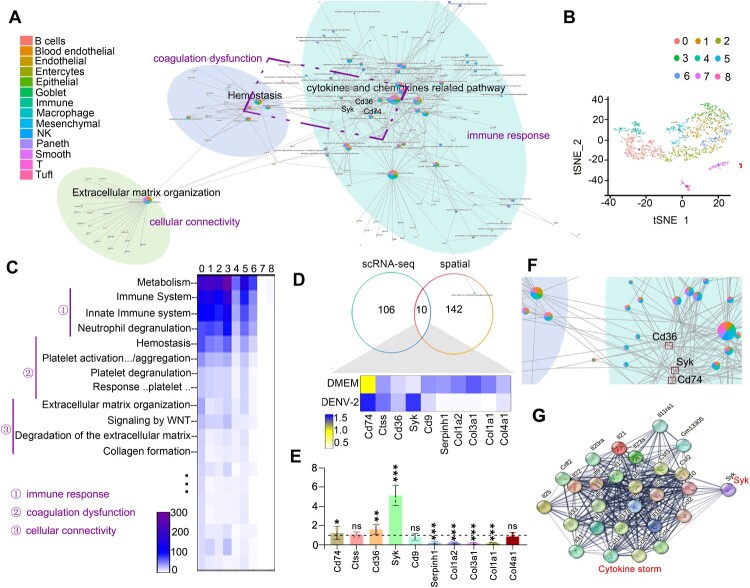
DENV-2 infection induces a Th2-type cytokine storm, leading to increased vascular permeability. (A) GO function enrichment analysis of DEGs among all cell types between the DMEM and DENV-2 groups. (B) Gene spots were divided into nine clusters ranging from 0 to 8 by tSNE map. (C) Reactome function enrichment analysis. Reactome function enrichment analysis of DEGs in clusters 0–8 were showed in heatmap. (D) The overlap analysis. The overlap analysis among the DEGs from the pathways co-related to platelet activation/aggregation, hemostasis, extracellular matrix organization between GO and reactome function enrichment analysis. (E) The mRNA level of target genes. (F) The key genes *CD36, CD74* and *Syk*. *CD36, CD74* and *Syk* linked the network of coagulation dysfunction and immune response. (G) The relationship between Syk and the production and release of cytokines showed by protein-protein interaction (PPI) network analysis.

Considering these subsets’ capacity to secrete inflammatory mediators, their aggregation may exacerbate immune-mediated intestinal hemorrhage through cytokine storm induction [5,39]. For mechanistic exploration, multi-dimensional functional annotation of DEGs was performed: GO analysis of scRNA-seq DEGs revealed three core functional modules – extracellular matrix (ECM) remodeling, hemostasis regulation, and cytokine/chemokine network modulation (Figure 6A and Figure S5). Cytokine modules were primarily driven by B cells, immune-related cells, macrophages, and T/NK cells; hemostasis functions correlated with B cells, endothelial cells, and smooth muscle cells; ECM remodeling involved coordinated actions of hemogenic endothelial, mesenchymal, and smooth muscle cells; Reactome pathway analysis of spatial transcriptomic DEGs from cluster cells further confirmed central pathways in immune dysregulation, coagulation abnormalities, and cell junction remodeling (Figure 6B,C and Figure S4O,P), corroborating GO findings. Cross-integration of spatial and single-cell DEGs identified 10 hub genes: *Col1a2*, *Col1a1*, *Col3a1*, *Col4a1*, *Serpinh1*, *Cd9* (downregulated), and *Syk, Cd74*, *Ctss*, *Cd36* (upregulated) (Figure 6D). The qPCR validation confirmed significant downregulation of *Col1a2, Col1a1, Col3a1, and Serpinh1,* with concurrent upregulation of *Syk, Cd74, and Cd36.* Notably, *Cd9, Ctss* and *Col4a1* exhibited no statistically significant differences (Figure 6E). Previous studies indicate that Syk promotes Th2-type inflammatory responses [40,41], while the roles of Cd74 and Cd36 in Th1/Th2 polarization bias remain controversial [42–45]. These genes are functionally associated with coagulation regulators [46]. In this study, we identified of Syk as a core node bridging immune responses (cytokine secretion) and coagulation/hemorrhage processes (Figure 6F), as well as the regulation role of Syk to cytokine storm network by protein–protein interaction (PPI) analysis (Figure 6G), substantiate the existing pathogenic hypothesis of DHF: Cytokine storms can directly disrupt endothelial tight junctions, significantly increasing vascular permeability and triggering hemorrhage with multi-organ dysfunction [35, 40–44]. Collectively, these data indicated that Syk drove the occurrence of DHF *via* regulating cytokine secretion and vascular endothelial dysfunction.

### Syk-mediated th2-polarized cytokine storm

**Figure 7.**
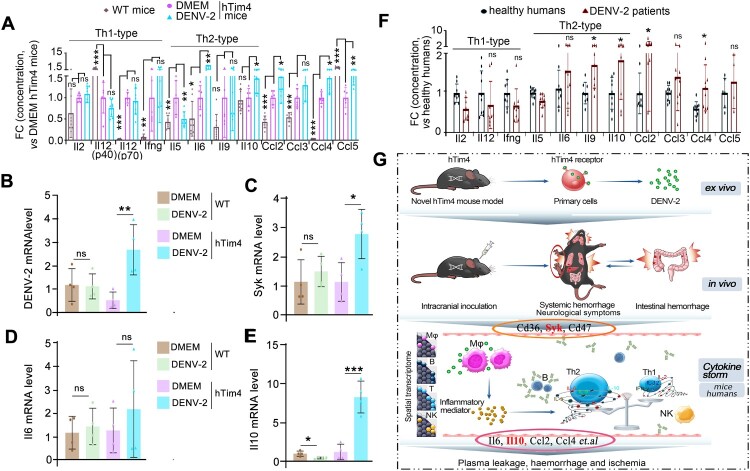
**The validation of conserved Th2 cytokine storm signatures across human and murine systems.** (A) Elevated levels of Th2-type cytokines and pro-Th2 chemokines were observed. Il2, Il5, Il6, Il9, Il10, Il12, Ifng, Ccl2, Ccl3, Ccl4 and Ccl5 were examined in DENV-2 infected hTim4 mice (n = 8), DMEM mice (n = 6) and wild-type C57BL/6 mice (WT, n = 8). (B-E) Validation of Syk driven Th2 bias by RT-qPCR. In the intestinal tissue form DENV-2 infected WT and hTim4 mice, the mRNA level of DENV-2, Syk, Il6 and Il10. (F) The cytokines bias in DENV-infected patients (n = 10) and healthy humans (n = 10). (G) The graphical abstract of our study. Data: mean ± SD. Statistical analysis: A-F: Two-sided unpaired Student's *t*-test. * *P*<0.05; **P<0.01; ***P<0.001. The exact *P* values were numerically documented in Table S4.

Intensive studies have elucidated that cytokine storms are a key factor causing DHF[1,3,8], and we revealed that Sky might regulate the secretion of cytokines (Figure 6G), and then we wanted to know the bias of cytokines. Initially, flow cytometry was employed to analyse serum cytokine profiles in hTim4 mice with/without DENV-2-infection. To eliminate potential interference from hTim4-induced immune responses [47], a parallel VSV infection control experiment was specifically designed (Figure S6A). Results demonstrated significant elevation of Th1 cytokines in both groups post-VSV infection, confirming viral factors rather than hTim4 as the primary driver of Th1 responses, thereby effectively excluding hTim4-related confounding effects on Th2 detection. The results showed that hTim4 mice exhibited marked Th2-polarized cytokine storm characteristics compared to DMEM controls: Il6 (5.3-fold increase, *P* < 0.01), Il10 (1.4-fold increase, *P* < 0.05), Ccl2 (1.48-fold increase, *P* < 0.05), and Ccl4 (1.44-fold increase, *P* < 0.05) were significantly upregulated (Figure 7A), while Th1 cytokines (I12–2, Il12p40/p70, Ifng) showed no statistically significant alterations [5,27]. To reveal the relationship between Sky gene expression and cytokine secretion, qRT-PCR validation revealed significant *Syk* upregulation in intestinal tissues of DENV-2-infected hTim4 mice (*P* < 0.05, Figure 7B, C and Table S2), accompanied by markedly elevated Il10 (*P* < 0.001, Figure 7E) and a trend towards Il6 increase (*P* = 0.4541, Figure 7D). In contrast, WT mice showed only marginal trends in *Syk* and Il6 expression, with significant Il10 downregulation (*P* < 0.05). scRNA-seq further delineated a Th2-characteristic molecular signature: significant upregulation of Il10, Ccl2, and Ccl5 (RANTES) alongside pronounced Ifng reduction (Figure S6B, C). Multi-omics data collectively confirmed Th2-type immune bias in hTim4 mice post-DENV-2 infection. To validate the clinical relevance of this Th2 bias, serum samples from a human cohort during the 2014–2015 DENV-2 epidemic in Southern China (including DF/DHF patients and healthy controls) were analysed. The human infection group displayed a Th2-dominant cytokine storm pattern highly consistent with the murine model (Figure 7F), establishing cross-species conservation of this phenomenon. Evidences from clinical observations also indicated that Syk activation promotes the secretion of proinflammatory mediators such as Tnfα and Il6, potentially triggering systemic cytokine storms [48]. Additionally, patients with DH demonstrate a shift towards Th2-dominant cytokine responses [28]. In conclusion, this study systematically elucidates that DENV-2 infection induces Syk-mediated Th2-polarized cytokine storms, driving further the occurrence of DHF, providing critical insights into its pathological association with vascular hyperpermeability.

## Discussion

This study systematically confirmed through *in vitro*, *ex vivo* and *in vivo* experiments that the hTim4 molecule is a key functional receptor for DENV-2 infection. The hTim4 transgenic mouse model demonstrated similar disease phenotype with DHF in terms of immune response and molecular mechanism (Figures 2–7), providing a novel animal model that is both scientifically rigourous and clinically relevant for the analysis of dengue fever pathogenesis, and may further be developed for antiviral drug screening or vaccine evaluation. This model mediates DENV-2 infection through the natural receptor hTim4, precisely reproducing the hallmark features of dengue infection observed in humans, including high viremia (Figure 2), atypical symptoms or microhemorrhage and inflammatory response in the brain parenchyma with clinical dengue encephalitis features (Figure 3), as well as systemic hemorrhage and DSS induced by disseminated intravascular coagulation (DIC) (Figure 4). The infection-induced plasma leakage (ALB exudation, Figure 4M) and decreased mesenteric blood perfusion pressure (decrease > 40%, Figure 4E-G and Table S3) are highly consistent with clinical DHF/DSS symptoms; the pathological features in the brain are highly consistent with the thalamus/basal ganglia injury pattern in human dengue-related encephalitis[10,49]. Compared with the currently recognized AG129 mouse model (deficient in interferon-α/β/γ receptors), hTim4 mice have several advantages: (1) They Retaining the complete type I/II interferon signalling pathway, overcoming the abnormal function of immune deficiency [23]; (2) Mediating DENV infection through a natural receptor precisely simulates the Th2-type cytokine response (Figures 5–7), consistent with the immune characteristics of severe clinical patients (IL-10↑/IFN-γ↓) [25–28, 50]; (3) Overcomes the Th1 immune bias of C57BL/6 wild-type mice [51] and the Th1/Th2 response damage caused by immune deficiency in AG129 [21,23], more closely resembling the immune response pattern in the human population.

Dengue fever is naturally transmitted through arthropod-borne routes [52], whereas this study employs I.C. inoculation (Figures 2–4), which has certain limitations. The I.C. route cannot fully replicate the entire complexity of mosquito-borne transmission infection, especially the potential impact of mosquito salivary factors mediating immune regulation on infection and the complex interaction among vector-host–pathogen. The core objective of this study is to reveal the pathological changes induced by DENV infection leading to DHF/DSS, rather than to simulate the entire natural infection history. It is worth noting that existing studies have shown that despite different routes (subcutaneous, intraperitoneal, intracranial), key pathogenic phenotypes (such as plasma leakage) can be induced in the AG129 mouse model [20, 22, 53–55]. This indicates that certain core pathogenic mechanisms (such as vascular leakage) may be intrinsic responses of tissues/hosts rather than absolutely dependent on specific initial infection routes, which partly supports the rationality of our use of the intracranial route to study the dengue hemorrhagic fever disease model. Secondly, I.C. inoculation effectively bypasses the peripheral immune surveillance mechanism, facilitating the study of early inflammatory cascade reactions; the induced neurological symptoms can systematically analyse the pathophysiology of the neurovascular unit, which would be masked in the multi-system dynamics of natural infection; it extends the window period of the disease process (Figure 2), which is conducive to studying the pathological mechanism of immune disorder-plasma leakage.

Clinically, dengue encephalitis is predominantly mild or asymptomatic [56,57], while severe dengue (e.g. DHF/DSS) is key fatal due to antibody-dependent enhancement (ADE) and cytokine storm-induced vascular leakage syndrome (shock, multi-organ failure) within 3–7 days post-infection (https://apps.who.int/iris/handle/10665/44188) [49]. Consistent with this, AG129 mice exhibit rapid mortality within 7 days due to immunodeficiency [23,53,54,58]. However, their pathological features (e.g. multi-organ hemorrhage) likely result from non-specific immune exhaustion triggered by ultra-high viral doses (10^4∼8^ PFU), which fundamentally differ from human immunopathology [23,53,54,58]. Such high doses may breach physiological immune barriers, causing non-target tissue damage and obscuring authentic virus-host dynamics. In addition, wild-type BALB/c (DENV-2) and C57BL/6 (DENV-3) mice develop severe encephalitis and brain tissue damage following I.C. inoculation [51,59,60]. whereas hTim4 mice display only mild encephalitis (Figure 3), suggesting their utility in modeling asymptomatic/mild clinical encephalitis.

Notably, while severe dengue (DHF/DSS) is more common in secondary infections, primary infections can also induce critical illness [49]. A clinical study of 344 pediatric cases showed comparable severe disease rates between primary (32.5%, 112 cases) and secondary infections (32.7%). Infants (≤1 year) exhibited the highest severe rate (64.7%), exclusively associated with primary infections; among fatal cases, 5 originated from primary and 2 from secondary infections [49]. These primary infection cases met the WHO 2009 criteria for severe dengue, including shock (pulse pressure ≤20 mmHg), severe plasma leakage, hemorrhage (gastrointestinal/intracranial), and organ failure, with mortality driven by circulatory collapse and multi-organ dysfunction rather than encephalitis (https://apps.who.int/iris/handle/10665/44188) [49]. These clinical findings converge with our research conclusions to substantiate the need for critical reappraisal of ADE in secondary infections as the predominant pathogenic mechanism underlying severe dengue manifestations. Notably, multifactorial determinants – including serotype-specific variations, virulence determinants of dengue strains, direct viral endothelial cytotoxicity, and host immune dysregulation – may collectively orchestrate the pathological progression towards severe disease outcomes in primary infections. Compared to the dengue fever disease model of AG129 model, the hTim4 mouse model is low-dose specificity, requiring only 50 PFU to trigger controlled inflammatory responses (e.g. IFN-λ-mediated antiviral signalling), avoiding high-dose-induced non-target tissue damage (Figure 2–4). And it Preserved natural immune pathways, faithfully reflecting virus-host equilibria (Figure 1). Extended disease progression window (50% survival: 12 days; 100% mortality: 18 days) enables phased analysis of Th2 polarization and vascular leakage mechanisms (Figures 2 and 7). Although AG129 mice rapidly replicate the 7-day lethal window, their immunodeficiency-driven multi-organ hemorrhage (a direct consequence of innate immune deficiency) diverges from clinically relevant immune-driven mechanisms (e.g. endothelial injury) [21,23]. As to adult wild-type mice, they were not susceptible to DENV [61,62], failed to develop hallmark symptoms of DHF/DSS even at high viral doses [51] and maintain an prolonged infection window (Figure 2N). In contrast, hTim4 mice recapitulate vascular leakage and organ damage at ultra-low doses, closely mirroring human pathology and providing a robust tool to transcend ADE limitations and explore novel strain-host interaction mechanisms. Future optimizations, including viral serotype/strain selection and alternative infection routes will enhance the clinical translatability of vascular leakage phenotypes. In summary, while hTim4 mice cannot fully replicate natural vector-borne transmission, they offer critical insights into primary infection-driven severe mechanisms of DHF/DSS.

Our study employed integrated scRNA sequencing and spatial transcriptomics to elucidate the molecular mechanisms driving immune dysregulation and hemorrhagic manifestations during DENV-2 infection. Our findings demonstrate that intestinal B cells, vascular endothelial cells, and immune-associated cells collectively orchestrate dysregulation of cytokine-coagulation crosstalk through coordinated modulation of prothrombotic factors (Col1a1, Col3a1) [46] and the Syk signalling network (Figure 5,6 and Figure S4–5). It is worth noting, the Syk molecular may plays key roles. Our results indicated that three core nodes emerged at the immune-hemostatic interface (Figure 6). Syk potentially mediates Th2-polarized cytokine secretion (Il-6/Il-10) [40,41], Cd36 enhances hematoma resolution via Il-10/Stat3 signalling [42,43] and Cd74 synergizes with MIF to amplify inflammatory cascades [44,45]. Experimental validation revealed Syk upregulation in DENV-2-infected murine tissues, correlating with Th2-skewed cytokine profiles mirroring clinical dengue cases (Figure 7 and Figure S6). The observed Syk-cytokine network interactions (Figure 6) suggest Syk could coordinately drive hemorrhagic pathogenesis through dual modulation of immune and coagulation systems. Notably, primary infection cohorts exhibited severe phenotypes pathologically analogous to ADE-associated secondary infections, with comparable epidemiological prevalence [49]. While our multi-omics data predominantly support direct cytokine storm-mediated endothelial injury – particularly through IL-10 overproduction – partial immunological overlap with ADE mechanisms was evident, including monocyte/macrophage activation and Il-10 dysregulation. Although classical ADE typically involves heterotypic reinfection via Fcγ receptor-mediated viral internalization and innate immunity suppression [63], we identified ADE-like immunological signatures in severe primary infections, implying alternative pathway being activated. In all, we propose hypotheses to explain the pathogenesis model: both primary infection-induced Th2 polarization and secondary infection-mediated ADE ultimately disrupt Syk-regulated cytokine-coagulation homeostasis, culminating in vascular leakage. These insights inform further a precision therapeutic framework: strategic Syk inhibition may rebalance Th1/Th2 responses to mitigate cytokine storms, while concurrent Cd36/Cd74 targeting could address coagulation abnormalities. Targeting Syk signalling pathway represents a possibly therapeutic strategy for dengue fever. Activation of Syk contributes to severe pathological processes, including vascular leakage and cytokine storm, by promoting the release of pro-inflammatory cytokines and excessive activation of immune cells. Inhibition of Syk may mitigate dengue-related tissue damage through regulation of the immune pathological response; however, its direct effects on viral replication require further verification. Currently, the clinically approved Syk inhibitor Fostamatinib is predominantly utilized for treating adult chronic immune thrombocytopenia (ITP) [64], with no studies exploring its application in dengue fever. Other investigational Syk inhibitors, such as Entospletinib and PRT062607, are primarily being evaluated in clinical trials for autoimmune diseases [65,66] and hematological malignancies [67,68]. Notably, PRT062607 has demonstrated potential for intervening in sepsis-induced heart dysfunction and multiple organ failure [69]. Based on existing research evidence, repurposing evaluations of Syk inhibitors or the development of next-generation compounds with enhanced pharmacokinetic properties and safety profiles could provide a breakthrough direction for this field. Given limited antiviral efficacy of single-pathway interventions, combinatorial regimens incorporating neutralizing monoclonal antibodies are warranted. Vaccine development must prioritize Th1-biased immunity to counter pathogenic Th2 polarization while circumventing ADE risks. By delineating the immune-hemostatic interactome, this work establishes a multi-target intervention paradigm for DHF management.

This study demonstrated that the Th2-biased cytokine storm pattern observed in hTim4 mice with DENV-2 infection, characterized by elevated levels of Il6 (**P = 0.0082), Il9 (*P *= 0.3965), Il10 (*P = 0.0168), was highly consistent with the serum profiles of clinical dengue fever patients (Figure 7). Notably, the Il10 levels and liver injury markers AST/ALT revealed a strong positive correlation [70]. Given that liver injury was associated with coagulation dysfunction, high Il10 expression was verified to closely link to adverse outcomes in both dengue fever and COVID-19 [71,72]. As reported, primary infection predominantly induced a Th1 immune response [73,74], whereas secondary infection markedly enhanced Th2 cytokine production [75,76]. This shift from Th1 to Th2 immunity might exacerbate plasma leakage and organ damage through endothelial dysfunction mediated by Il6 and Il8 [77,78], thereby promoting the progression of severe dengue manifestations, such as DHF and DSS [79,80]. Previous studies exhibited divergent perspectives on the temporal dynamics of Th2 responses: some indicated that these responses predominantly occurred during the recovery phase [81], whereas others highlighted their persistence throughout the acute and defervescence phases [77]. Our findings not only support the association between Th2 polarization and the progression of severe dengue [77,82], but also offer a novel framework for validating traditional disease-stage immune models. This research innovatively developed an hTim4 mouse infection model to comprehensively investigate the molecular mechanisms underlying DENV-2-induced hemorrhagic manifestations resembling DHF/DSS. Utilizing a robust experimental framework, the study integrated intracranial viral challenge, stringent virus control experiments, cross-species clinical validation, and multi-omics analysis. These approaches collectively revealed that DENV-2 infection triggers Syk-mediated Th2-polarized cytokine storms, which intensify vascular hyperpermeability and plasma leakage (Figure 7G). The experimental design adhered strictly to control principles, while data analysis incorporated multiple corrections to ensure the scientific rigour of the findings. The strong correlation between the model system and clinical observations underscores the reliability of hTim4 mice in mimicking the immune response to DENV infection, providing a solid theoretical basis for future mechanistic studies.

## Materials and methods

### Mice, ethics and serum samples

Wild-type C57BL/6J mice and hTim4 genetically modified mice were bred by the Institute for Laboratory Animal Resources, NIFDC, and maintained in a SPF facility. These mice, aged between 3–11 weeks, were randomly assigned to experimental groups. The age of all mice remained consistent throughout each individual experiment. Virus inoculation and dissection procedures were performed in an ABSL-2 facility under anesthesia induced and maintained with Zoletil 50 and xylazine. All studies received approval from the Institutional Animal Care and Use Committee at the Institute for Laboratory Animal Resources, NIFDC (2019-B-16, 2024(B)027). The serum assay of dengue patients was reviewed by the Institutional Ethics Committee, Yunan Institute of Parasitic Diseases (No.03 - “Technical Cooperation Research on Prevention and Control of Emerging Vector-borne Infectious Diseases and Rapid Response Treatment in Belt Road Area”).

### Establishment of htim4 genetic modified mice

The human Tim4 (hTim4) gene was precisely inserted into the Tol2 vector. Briefly, a CAG promoter and hTim4 gene cDNA (NM_001146726), followed by an EGFP sequence, were synthesized by Sino GenoMax and cloned into Tol2 vector (donated by BIOCYTOGEN). Tol2 transposase mRNA was transcribed *in vitro* from the plasmid pT7-Tol2 using mMESSAGE mMACHINETM T7 Transcription Kit (Ambion, AM1344), then add A using Poly(A) Tailing Kit (Ambion, AM1350) and purified by RNeasy Mini Kit (QIAGEN, 74104) according to manufacturer’s instructions. Subsequently, both Tol2 transposon DNA (30 ng) and mRNA (50 ng) were micro-injected together into C57BL/6J mouse zygotes which were further transplanted into pseudo-pregnant mice to generate founder mice (Table S1). Genotyping of offspring was conducted to screen hTim4 gene positive founder mice (Table S2).

### Fluorescence detection of htim4 mice *in vivo*

The neonatal mice aged 3–5 days, genotyped as hTim4 positive, were utilized for *in vivo* fluorescence detection using the IVIS Spectrum CT imaging system. The expression of hTim4 was quantified by measuring EGFP immunofluorescence through radiant efficiency analysis.

### RNA-Seq

The total RNAs from lung tissues were isolated using the RNeasy Plus Universal Mini Kit (Qiagen, 73404) and assessed for quality (OD260/280 = 1.8∼2.2, OD260/230 ≥ 2.0, RIN ≥ 8, 28S:18S ≥ 1.0, > 10 μg). Subsequently, they were subjected to treatment with the TruSeqTM stranded total RNA Kit (Illumina, 20020596) for transcriptome strand library preparation. Initially, ribosomal RNA was depleted using the Ribo-Zero Magnetic kit (Illumina, 20040526), followed by fragmentation of the remaining RNAs. First-stranded cDNAs and double-stranded cDNAs were then synthesized and purified accordingly. Each blunt fragment of the resulting double-stranded cDNAs had a single “A” nucleotide added at its 3′ end as a handle for ligation with multiple indexing adapters at both ends of the ds cDNAs. Target fragments ranging from 300 to 420 bp in size were selected and amplified before quantification using a TBS380 mini fluorometer (Tubner Biosystem). Finally, the constructed RNA-seq library was sequenced on an Illumina HiSeq xten//NovaSeq6000 platform by Novagen (TianJin, China). All procedures strictly adhered to the instructions provided by Illumina.

### Identification of differential expressed genes

The differential expression of genes (DEGs) was analysed between wild-type (WT) mice (used as a reference group) and hTim4 C57BL/6J mice. Subsequently, the clean reads were aligned to the murine reference genome using HIASAT software in orientation mode. The TPM method was employed to calculate the expression level of each transcript, while RSEM was used for gene abundance quantification. DESeq2/DEGseq/EdgeR with adjusted *P*-values were utilized to determine whether a gene exhibited differential expression. Genes with an adjusted *P*-value ≤ 0.05 (as determined by DESeq2 or EdgeR), along with fold changes > 1 or < −1 for differentially expressed mRNAs (DEmRNAs), were considered as exhibiting significant differential expression between lung tissues of WT and hTim4 mice.

### Reactome functional enrichment analysis

To elucidate the functional profile of differentially expressed genes (DEGs), we conducted Reactome pathway enrichment analysis for gene lists. The top five pathways enriched with DEGs were selected for further investigation. Violin diagrams were employed to present the top ten genes in each pathway.

### Determination of tissue distribution of htim4 mRNA expression

The hTim4 mice were anesthetized using Zoletil 50 plus xylazine. Subsequently, the heart, liver, spleen, lung, kidney, intestine, brain and reproductive organs were harvested and weighed before being homogenized through bead dissociation with a tissue grinder (Qiagen). Total RNA levels were extracted using canonical precipitation with trizol, chloroform, iso-propanol and quantified via quantitative reverse transcriptase PCR (qRT-PCR) following the instructions provided in the RT reagent Kit (Takara, RR047) and qPCR kit (Takara, RR820). Validation of dengue RNA was performed by qRT-PCR utilizing DENV-2 primers (Table S2).

### Flow cytometry

Blood samples were collected from the inner canthus vein of mice in tubes containing 1% EDTA (0.5M/L). T, B, and NK cells in the peripheral blood were labelled using corresponding antibodies (BioLegend), including anti-Mouse CD45 antibody, anti-Mouse CD19 antibody, and anti-Mouse CD49b antibody. The BD LSRII flow cytometer and FACS Diva 6.0 Software were employed for detection and quantification.

### Blood routine examination

Blood samples were collected from the inner canthus vein of mice using EDTA-containing tubes, and blood routine examinations were performed with a Hematology analyer (Mindary, BC-5000-VET) according to the manufacturer's instructions.

### Cells and viruses

Vero cells were cultivated at 37°C in the minimum essential medium (MEM) complemented with 5% fetal bovine serum (FBS). Aedes albopictus C6/36 cells were grown at 28°C in RPMI 1640 medium enriched with 10% FBS. The DENV-2 (Tr1751 strain) utilized for the challenge model was offered by Professor An Jing from Capital Medical University. The virus propagated in C6/36 cells and was stored in a −80°C freezer. The titer of the virus (10^4^ PFU/ml)was ascertained by plaque assays on Vero cells.

### Absolute quantification of dengue genome copies

In our study, absolute RT-qPCR was conducted to quantify the viral load. Initially, a standardized plasmid containing a chemically synthesized 156 bp fragment from protein E of Tr.1751 genomic DNA oligonucleotide inserted into the pET-32a+ plasmid vector was generated. The standard quality control sample was diluted in a 10-fold gradient, and after DNA extraction, real-time qPCR was performed. Subsequently, the concentration of viral genome copies was calculated using the formula (genomic copies/μl = (6.02 × 10^23^) × (ng/μl × 10^−9^) ÷ (RNA/DNA length × 340/660)). “/”represents “either”. A standard curve based on the copy number concentration of each sample and its corresponding CT value was plotted, yielding a linear equation: Y = −3.141 lgX + 36.077, R^2^ = 0.9971. Prior to RT-qPCR detection, the test sample underwent treatment with RNase A at a concentration of 100 μg/mL to eliminate naked viral RNAs. The CT value obtained for the test sample was then used in equation Y to determine the copy number concentration and subsequently assess the viral load.

### Prothrombin and activated partial thromboplastin time test

The blood of anesthetized mice was collected in tubes containing 1/10 sodium citrate (10^9^ mM). Anticoagulation was achieved by centrifuging at 3000 rpm for 10∼15 min, followed by removal of platelets and collection of the resulting supernatant. Throughout the experiment, all reagents were halved in quantity. The assays were performed according to the protocols provided with the prothrombin (PT) test kit (SolarBio, BC8081) and activated partial thromboplastin time (APTT) test kit (SolarBio, BC8080).

### Primary liver and spleen cells culture

Primary hepatocytes were isolated using the two-step collagenase perfusion technique. Briefly, mice aged 6–13 weeks were anesthetized, and a combination of D-HBSS medium and collagenase IV enzyme was introduced into the circulatory system *via* the portal vein to perfuse the liver. The resulting hepatocytes were then purified through filtration with a 70 μm filter followed by centrifugation at 50 g for 2 min. Subsequently, they were cultured in William's medium supplemented with FBS, sodium pyruvate, HEPES, Insulin-Transferrin-Selenium, Dexamethasone, and Glutamax at a temperature of 37°C under a CO_2_ concentration of 5%. Primary spleen cells were obtained through grinding followed by purification using a 40 μm filter and centrifugation at 500 g for 5 min. These cells were also cultured in William's medium supplemented with FBS at a temperature of 37°C under a CO_2_ concentration of 5%.

### Animal experiment

The mouse infection experiments with dengue virus were conducted under ABSL-2 conditions. hTim4 mice were anesthetized using Zoletil 50 plus xylazine. The mice in the experimental group were intracerebrally inoculated with DENV-2 (strain Tr.1751) at dose of 50 PFU (corresponds to 10^3^ TCID_50_/ml 30 μl), 100 PFU (corresponds to 10^4^ TCID_50_/ml 30 μl) and 500 PFU (corresponds to 10^5^ TCID_50_/ml 30 μl), while the control group received intracerebral inoculation with DMEM. The viral volume administered was approximately 30 μL (1.67×10^3^ PFU/ml). The survival time, weight, and clinical score of the mice were monitored for a period of 21–28 days.

### Viral load of tissues

DENV-2-infected hTim4 mice were anesthetized using a combination of Zoletil 50 and xylazine. Blood samples (100 μl) were collected *via* orbital blood extraction at 2, 4, 6, and 8 dpi. Subsequently, RNA was extracted from the blood using RNA iso-Plus (Takara 9109). The brains and intestines were harvested, weighed, and homogenized with bead dissociation using a tissue grinder (Thermofisher). The RNAs were quantified by qRT-PCR for absolute quantification of dengue genome copies.

### Evans blue staining

The quantification of vascular leakage was performed using Evans blue staining. Briefly, each mouse received an intravenous injection of 150 μl of Evans blue dye (0.5% in PBS) at 8–11 dpi. For hTim4 mice, anesthesia was induced with Zoletil 50 plus xylazine and perfusion with PBS was conducted after a 2-hour interval. Subsequently, the brain, lung, and intestine were collected for extraction and dying using formamide (F7503, Sigma).

### Clotting time/blood drops

The hTim4 mice were anesthetized using a combination of Zoletil 50 and xylazine. Subsequently, the distal portion of their tails was amputated, and the subsequent hemostatic response was evaluated by quantifying the number of blood droplets and measuring clotting time.

### High resolution laser speckle contrast imaging (HR-LSCI)

After administering abdominal anesthesia, mice were positioned in a prone position and secured to the mouse fixator. Following alcohol disinfection, a longitudinal incision was made on the scalp to fully expose the skull. The periosteum was then separated, while keeping the skull moist with normal saline solution. Similarly, after alcohol disinfection, a longitudinal incision was made on the abdomen skin to fully expose the mesentery and intestinal tissue. Normal saline solution was used to maintain moisture during this process. Vessels and blood flow in mice were captured using an HR-LSCI laser speckle blood flow imaging system (LSI BFI PLUS, LogiScience Technology Co., Ltd，China). Parameters were set according to those specified in the HR-LSCI system settings section below. The system utilized a 780 nm semiconductor sweep laser as its light source with an exposure time of 5 min. Reflected light was collected by an sCMOS camera at a recording rate of 1 s/frame for 3 min. Perfusion information was converted into two-dimensional perfusion images using HR-LSCI imaging algorithm and LSI HMC motion correction algorithm, followed by image processing and analysis conducted through the built-in LSI BFI PLUS image software.

### Hematoxylin–Eosin (H&E) staining and examination

The brain, lung, and intestine tissues from hTim4 mice and DMEM mice at 10–18 dpi were fixed in formalin, embedded in paraffin, and subsequently transferred to 70% ethanol for preservation. Each tissue sample was placed in processing cassettes, dehydrated through a sequential alcohol gradient, and finally embedded in paraffin wax blocks. Prior to immunostaining, 3 μm-thick tissue sections were dewaxed using xylene, rehydrated with decreasing concentrations of ethanol, and washed with PBS. Subsequently, the sections were stained using an H&E staining kit (Leagene; DH0003). The stained sections were scanned using a NanoZoomer scanner (HAMAMATSU; C13210-01) and previewed utilizing the Nano Zoomer Digital Pathology software (NDP.viewer).

### Immunohistochemistry examination

The hTim4 mice and DMEM mice were selected to assess the expression of specific markers. The intestinal tissue paraffin sections were dewaxed using xylene and a serial alcohol gradient, followed by incubation in 3% H_2_O_2_ for 15 min. Antigen retrieval was performed by boiling the sections in citrate buffer (pH 6.0). Subsequently, the sections were blocked with fetal bovine serum, and primary antibodies including CD31 (1:2000, rabbit, abcam, ab182981) were incubated overnight at 4 °C. After washing with PBS, secondary antibodies (1:250, servicebio, GB23303) were incubated for 1 h at 37 °C before sealing with glycerin. Panoramic scanning of the sections was conducted using a 3DHISTECH scanner (P250, FLASH). Capillary and microvessel density and diameter measurements were obtained from electron microscope images.

### Confocal assays

The intestinal tissue paraffin section was dewaxed using xylene and a serial alcohol gradient, followed by incubation in 3% H_2_O_2_ for 15 min. Antigen retrieval was performed by boiling with citrate buffer (pH 6.0). After blocking with fetal bovine serum, the following primary antibodies were incubated overnight at 4 °C (Table S5): DENV-2 NS1 (ab41616, abcam), CD31 (ab182981, abcam), villin (ab97512, abcam), murine albumin (ALB, SAB3500217, sigma) and VE-cadherin (ab205336, abcam). The secondary antibodies Donkey Anti-Rabbit IgG H&L-Alexa Fluor 647 (ab150075) and Goat Anti-Chicken IgY H&L-Alexa Fluor 555 (ab150170, abcam), Goat Anti-Mouse IgG H&L-Alexa Fluor 568 (ab175473, abcam) and Goat Anti-Mouse IgG H&L-Alexa Fluor 594 (ab150160, AbCam) were then incubated for one hour at 37 °C before examination under a confocal fluorescent microscope (Zeiss LSM900).

### Single-Cell RNA sequencing (ScRNA-seq) and data preprocessing

The fresh intestines of hTim4 and DMEM mice were applied for ScRNA-seq. The ScRNA-seq and data preprocessing were conducted by Beijing Capital Biotechnology Co., Ltd. The single-cell 3′ Library and Gel Bead Kit V3.1 provided by 10x Genomics (10x Genomics, 1000121) were used, along with the Chromium single-cell G-Code Kit (10x Genomics, 1000120), to process a cell suspension containing 300–600 live cells/mL, cell viability >85%, cell diameter: 5–40μm, cell clumping rate <20% and nuclear rate >70% as determined by Count Star. The suspension was loaded onto the 10x Genomics Chromium single-cell controller following the manufacturer's protocol in order to generate single-cell bead suspensions. Specifically, individual cells were suspended in PBS with 0.04% BSA, with approximately 11,000 cells being added to each channel with the goal of about 10,000 active cells. These active cells were captured and lysed to release RNA which was then barcoded and reverse transcribed in each GEM. The reverse transcription process was carried out in the Bio-Rad S1000TM Touch thermal cycler at 53°C for 45 min followed by incubation at 85°C for an additional five minutes before storage at 4°C. Subsequently, the generated cDNA underwent amplification and quality assessment using the Qubit3.0 Fluorometer and Agilent 2100 Bioanalyzer Tape Statio system (Agilent Technologies). The ScRNA-seq library was prepared using the Single Cell 3′ Library and Gel Beads Kit V3.1, and subjected to sequencing on the Illumina Novaseq 6000 platform with paired-end (PE) reads of 150 bp in length. The sequencing depth reached a minimum of 50,000 reads per cell. For the quality-controlled data by FastQC (v0.11.2), we utilized the Cell Ranger software's (V6.0) cellranger count module for alignment, filtering, barcode counting, and UMI counting to generate feature barcode matrices and identify clusters. Furthermore, cells with fewer than 200 genes or lower than 99 UMI, as well as those with mitochondrial gene proportions exceeding 25%, are filtered using Seurat (v4.0.0). Principal component analysis (PCA) is then performed for dimensionality reduction, followed by visualization using TSNE and UMAP. Next, the Single R uses reference transcriptome data for pure cell types to infer the origin of each individual cell, thereby performing unbiased cell type identification on single cell RNA sequencing data. For humans, use Blueprint_Encode or HPCA was used, and for mice, ImmGen or Mouse.RNAseq. The cell types annotated by Single R (https://bioconductor.org/packages/devel/bioc/html/SingleR.html) are manually corrected using literature-reported cell-specific Marker genes. Differential genes (DEGs) are calculated for each cell type, and then enrichment analysis is performed using GO (Gene Ontology, http://geneontology.org/), KEGG (Kyoto Encyclopedia of Genes and Genomes), and Reactome (http://www.reactome.org/) databases, and the significance level of pathways is calculated using Fisher's test, with the goal of identifying gene sets that are enriched in the cell type of interest. The results were visualized using R package.

### Spatial sequencing

Briefly, the intestines of hTim4 mice were embedded using SAKURA (4583) and subsequently frozen at −80 °C. The embedded tissue blocks underwent RNA integrity assessment by Agilent 2100, ensuring a RIN value greater than 7. For construction of the sequencing library, the Visium Spatial Gene Expression Slide & Reagent kit from 10 × Genomics (PN-1000184) was employed. Each tissue slide was meticulously positioned on a Capture Area within a Visium gene expression slide, followed by staining with Hematoxylin and Eosin (H&E), imaging, and permeabilization. During permeabilization, mRNA molecules were released and specifically bound to oligonucleotides that had been pre-coated on the Capture Area. Subsequently, reverse transcription was performed and sequencing libraries were prepared according to the manufacturer's protocol. The resulting raw FASTQ files and pre-existing histology images were processed using Space Ranger (v1.2.0, 10 × Genomics) with default parameters. The filtered gene-spot matrix and fiducial-aligned low-resolution image were utilized for downstream data analyses. Gene expression normalization, dimensionality reduction, spot clustering, and differential expression analysis were conducted using the Seurat package. Spots with less than 100 detected genes were excluded from further analysis. Normalization across spots was achieved using the built-in SCTransform tool, followed by selection of 3000 highly variable genes for principal component analysis (PCA). Spot clustering involved building a graph based on the first 20 principal components (PCs), which was then segmented at a resolution of 0.5 units. Differential gene expression analysis for each cluster was performed *via* Find All Markers function utilizing non-parametric Wilcoxon rank sum test criteria. Genes exhibiting fold change >2 and adjusted *P*-value <0.05 were considered significantly differentially expressed genes (DEGs). Enrichment testing for candidate gene sets employed the cluster Profiler R package based on hypergeometric distribution principles. Pathways with corrected *P*-values below 0.05 were deemed significantly enriched, while reactome enrichment analysis was employed to profile DEG functions.

### Cytokine testing and analysis

The serum was obtained by centrifuging the collected blood samples from hTim4 mice and DENV-2 infected patients at 10,000 rpm. Cytokine concentrations in the serum were quantified as pg/mL using the MSD (Meso Scale Discovery) method with the Bio-Plex 200 system and Bio-Plex Pro Mouse Cytokine 23-plex Assay (M60009RDPD, Bio-Rad, USA), and Bio-Plex Pro Human Cytokine 27-plex Assay (#M500KCAF0Y, Bio-Rad, USA) following the instruction manual (10014905, Bio-Rad, CA, USA).

## Statistical analysis

The statistical analyses were conducted using Prism V.8.0 and V.12.0 (GraphPad, La Jolla, California, USA). All data are presented as means ± standard deviation (SD). Statistical significance between two groups was determined using two side unpaired Student's *t*-test, One-Way ANOVA, and Two-Way ANOVA. Significance levels were denoted as follows: *P < 0.05; **P < 0.01; ***P < 0.001; ****P < 0.0001; ns: not statistically significant). Most experiments were performed at least three times to ensure reproducibility of the key findings.

## Author contributions

Conceptualization, F.C.F. and W.Y.C.; Writing – Original Draft, F.C.F., W.Y.Y. and Y.Y.S.; All experiments, Y.Y.S., W.Y.Y, Y.W., C.H.H. and X.R; Mouse modeling construction, L.S.S, Z.H.Y., G.W.D, W.X. and C.Y.; Mouse modeling experiments, Y.Y.S., X.R. and W.Y.Y.; Viral experiments, Y.W., L.S.N. W.Y.N. and L.C.; Functional experiments, Y.Y.S. and W.Y.Y; Pathological diagnose, Q.Z., Y.Y.W, L.Z. and W.S.L; Sequencing analysis, C.H.H.;Other supports, H.W.J., F.R., and Z.H.N.; Statistical analysis, Y.Y.S.; Writing-Review & Editing, W.Y.Y, Y.Y.S., F.C.F. and W.Y.C.; Resources, A.J. and W.W; Supervision, F.C.F. and W.Y.C. All authors have read and approved the article.

## Supplementary Material

Revised_supplemental_Table_and_Figure-clean.doc

## Data Availability

The data that support the ﬁndings of this study are available from the cor-responding author upon reasonable request. The data presented in the study are deposited and accessible through “https://www.ncbi.nlm.nih.gov/sra/”, RNA sequencing accession number PRJNA1090927. Spatial transcriptome sequencing accession number PRJNA1090978. The scRNA-seq accession number PRJNA1154714.
