# Supplementary material for: A novel immunocompetent transgenic mouse model of DHF reveals Syk-mediated Th2-polarized cytokine storm as a key driver of vascular leakage
Source: Emerg Microbes Infect. 2025 Jul 7;14(1):2531178. doi: 10.1080/22221751.2025.2531178 (PMC12291195; doi:10.1080/22221751.2025.2531178)
Supplement: Revised_supplemental_Table_and_Figure-clean.doc [file TEMI_A_2531178_SM7489.doc]

**Supplementary Data for A Novel Immunocompetent Transgenic Mouse Model of DHF Reveals Syk-mediated Th2-Polarized Cytokine Storm As A Key Driver Of Vascular Leakage**

**Supplemental Data:**

**Fig. S1**.Immune, hematological indices and blood physiology of hTim4 mice.

**Fig. S2**.Identification of optimal conditions for DENV-2 infection.

**Fig. S3.** The hTim4 mice infected with DENV-2 exhibited neurological manifestations and organ hemorrhage.

**Fig. S4**. The GO enrichment analysis of DEGs from scRNA-seq showed by network mapping.

**Fig. S5.** Molecular bioinformatics signatures of intestinal bleeding in mice with DHF.

**Fig. S6.** The cytokine levels in hTim4 mice.

**Supplemental Tables:.**

**Table S1**. The breading summary of hTim4 founder mice.

**Table S2**. Primers used in this study..

**Table S3.** The Pathological score and perfusion pressure in healthy, moderate and severe state.

**Table S4** The exact P values in this study.

**Table S5.** The materials in this manuscript.

**Table S6**. The criteria for “slow action", “limb paralysis” or "ataxia

**Table S7.** The criteria for pathological score.

Other Supplementary Material for this manuscript includes the following:


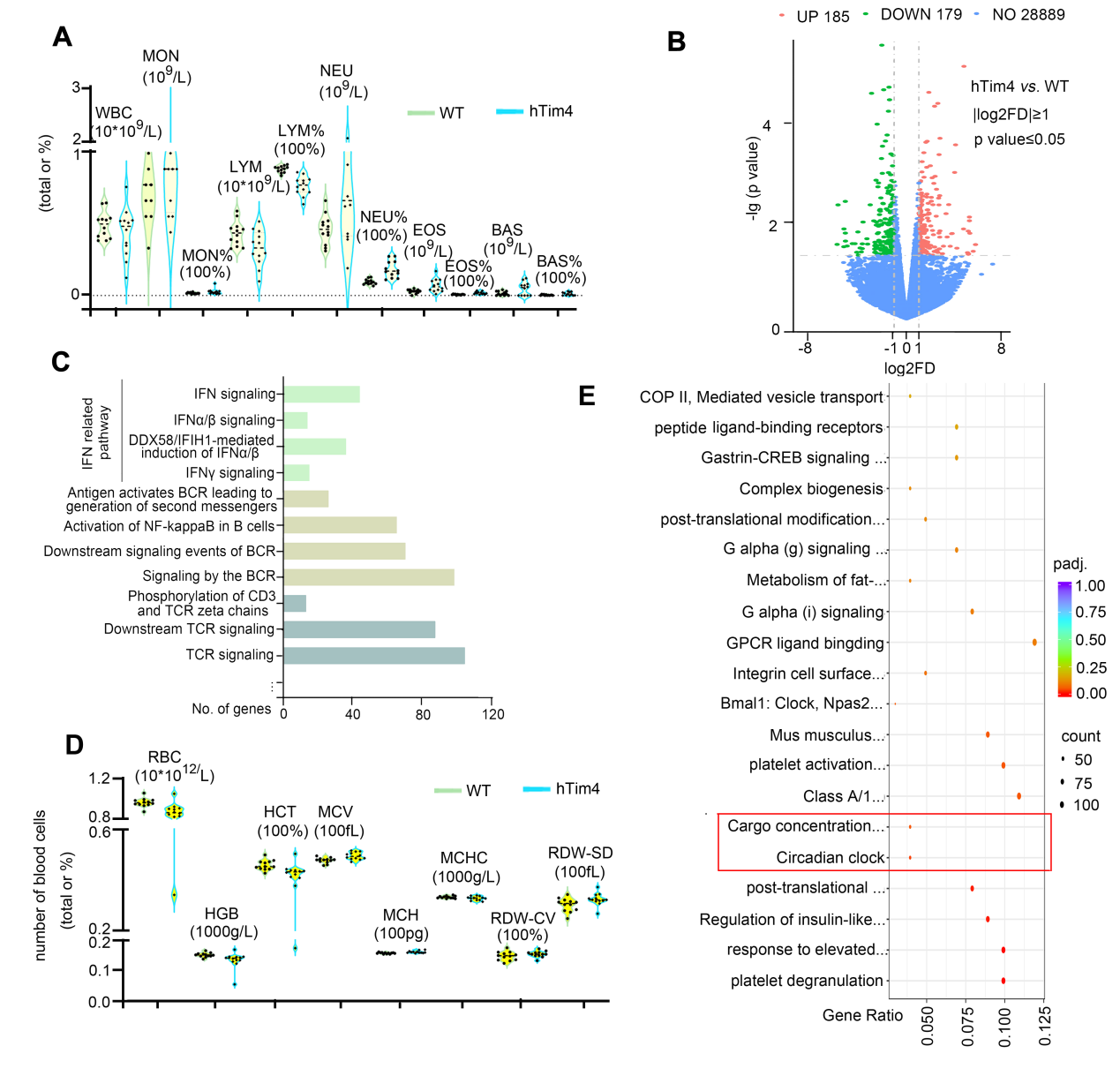


**Fig. S1 Immune, hematological indices and blood physiology of hTim4 mice.** (A) The counts of white blood cells (WBC), monocytes (MON), lymphocytes (LYM), neutrophils (NEU), eosinophils (EOS) and basophils (BAS) showed no significant difference between wild-type C57BL/6 and hTim4 mice. (B,C) IFN-related function. Total RNAs extracted from lung tissues were subjected to transcriptome strand library preparation for gene expression profiling. Volcano plots revealed only 1.21% differential gene expression between the hTim4 group (n=3) and WT group (n=3) for all murine genes analyzed (B). Reactome functional enrichment analysis showed normal IFN signaling pathway in all murine genes (C). (D) blood routine. The counts of red blood cells (RBC), HGB, HCT, MCV, MCH, MCHC, RDW-CV and RDW-SD also exhibited no significant difference between WT and hTim4 mice. (E) Reactome enrichment analysis was performed on the DEGs identified from lung tissues' RNA-seq data. Only cargo concentration and circadian clock pathways were found to be significant (red box). These findings suggest that overexpression of the hTim4 gene does not impact blood vessel fragility. Data: mean ± SD. Statistical analysis: A, D: unpaired Student's *t*-test; ns, not significant.


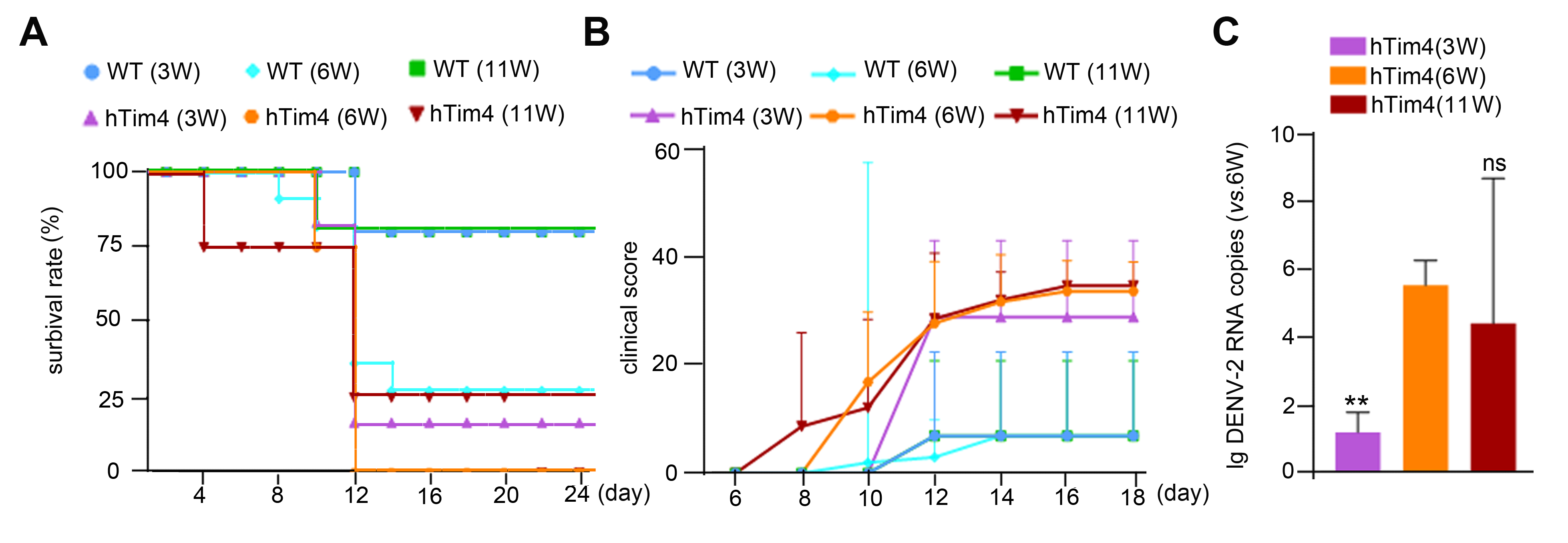


**Fig. S2 Identification of optimal conditions for DENV-2 infection.** C57BL/6 mice were intracranially injected with DENV-2 at a concentration of 500 PFU and divided into 6 groups: X-week-old wild-type (WT, XW) and hTim4 transgenic mice (hTim4, XW). (A) Survival curves at different ages. Survival curves show the survival rate after DENV-2 infection on days 4, 8, 12, 16, 20 and 24. (B) Clinical scores at different ages. Clinical scores at different ages indicate disease severity in infected mice on days 6, 8,10,12,14 ,16 and18. (C) Blood viremia at different ages. Detection of DENV-2 RNA in blood samples from infected mice confirms viremia. Statistical analysis: A,B, One-way ANOVA. C: unpaired Student's *t*-test. * *P*<0.05; ** *P*<0.01; *** *P*<0.001.

**Fig. S3 The hTim4 mice infected with DENV-2 exhibited neurological manifestations and organ hemorrhage.** Six-week-old hTim4 mice were intracranially injected with DENV-2 at a dose of 50 PFU (DENV-2 group, n=44), while the control group received DMEM infection (DMEM group, n=22). (A,B) Multi-organ bleeding. (C) HE staining of lung from DENV-2-infected hTim4 mice. Pathological features of lung hemorrhage. bleeding (+), pulmonary hemosiderosis (▲), and edema (★). (D-H) Blood coagulation function characteristics after DHF induced by viral infection. Clotting time prolonged (D) and blood drops increased (E) at 8 dpi in the DENV-2 group compared to the DMEM group. Biochemical markers including MCV levels (F), WBC (G), NEU (H) were evaluated. (I) In the moderate group, noticeable bleeding was observed in the DENV-2 group, indicated by the assigned mouse codes (number #). In the severe group, intestinal tissue exhibited cessation of bleeding and gangrene. Data are presented as mean ± SD. Statistical analysis: D,E,F-H: Unpaired Student's t-test. *P<0.05; **P<0.01; ***P<0.001.


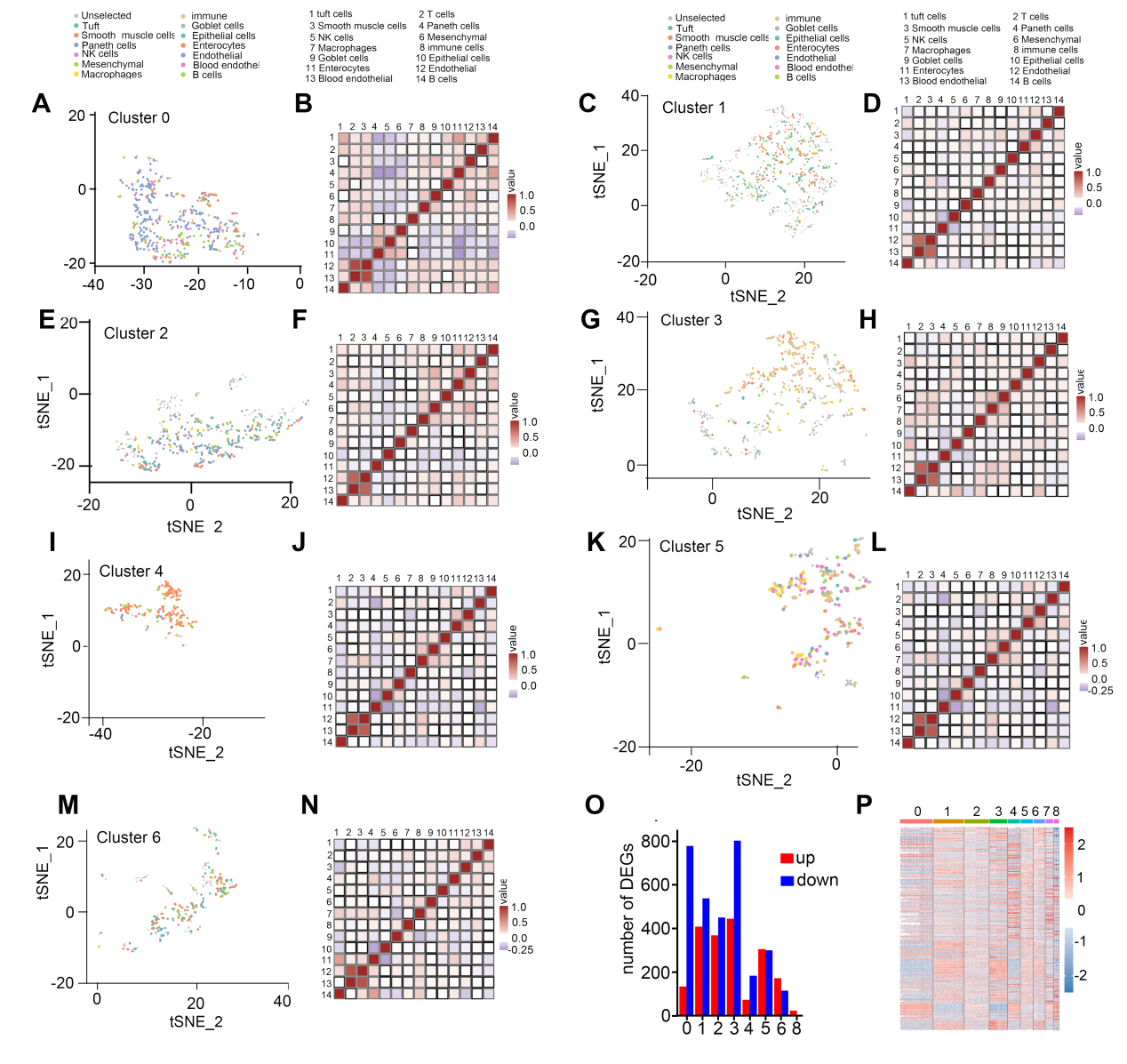


**Fig. S4 Molecular bioinformatics signatures of intestinal bleeding in mice with DHF**. (A-N) Spatial distribution and cell types ranging from cluster 0 to 6 are depicted. Fresh intestine tissues from the DMEM group (n=3) and DENV-2 group (n=3) were simultaneously frozen and embedded in optical cutting tissue (OCT) compound using liquid nitrogen. A 10 μm frozen tissue section was placed on one of the visium gene expression slide capture areas on a slide. Bright-field images were acquired following the Spatial Transcriptomics procedure, and sequencing libraries based on Novaseq PE150 were constructed using the visium Spatial Gene Expression Slide & Reagent kit (10 × Genomics). The expressions of sequencing libraries underwent normalization, dimensionality reduction, spot clustering, and differential expression analysis using Seurat software. tSNE mapping visualized the expression and distribution of all gene spots ranging from cluster 0 to 6 (A-N), while the heatmap demonstrated the correlation between spatial location and cell types (A-N). (O) The number of DEGs across clusters 0 to 8. (P) The expression levels of DEGs across clusters 0 to 8 by heatmap.


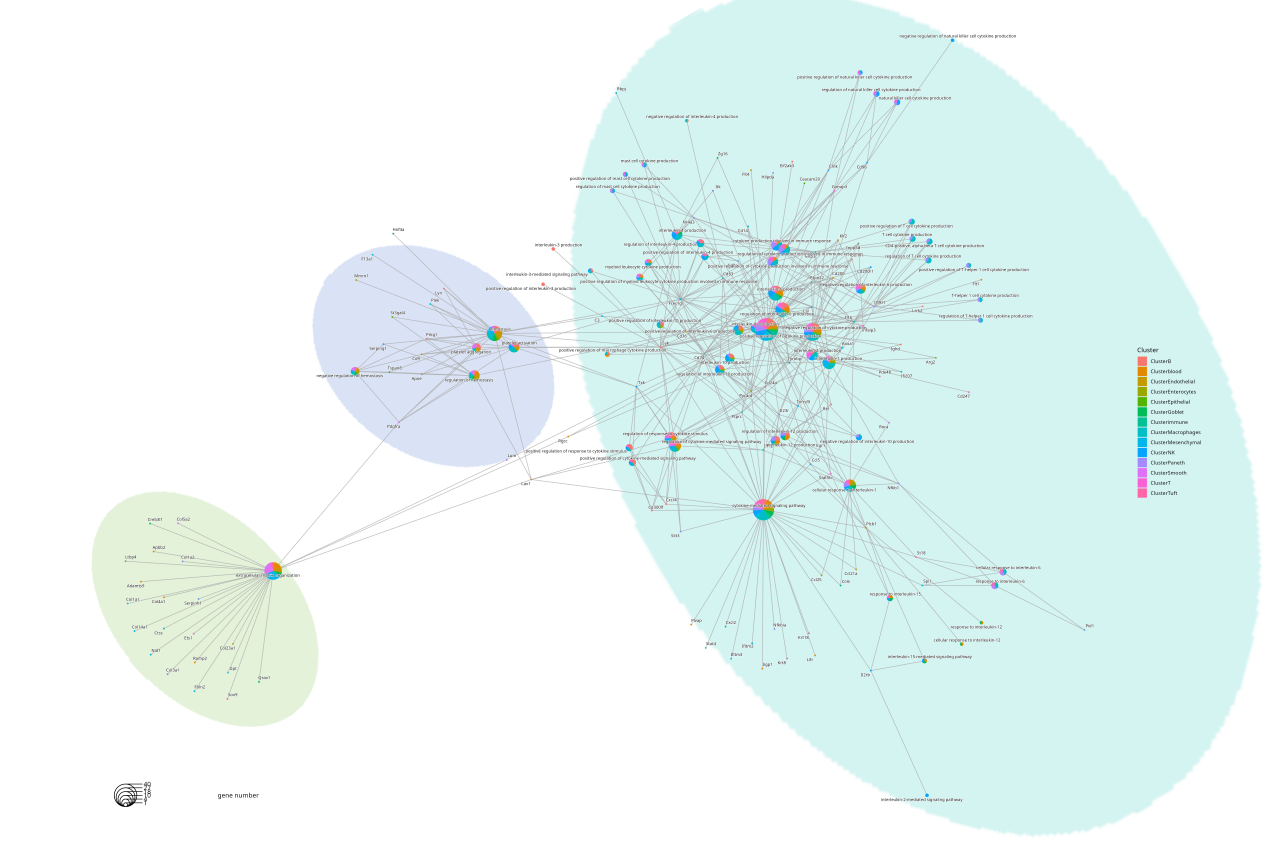


**Fig. S5 The GO enrichment analysis of DEGs from scRNA-seq showed by network mapping.**

**Fig. S6 The cytokine levels in hTim4 mice.** (A) The 6-week-old C57BL/6 (WT) mice and hTim4 mice were randomly divided into 4 groups with 12 mice in each group, respectively. 100 μl VSV (1x10^8^ PFU) were nasal inhabited into each mouse (VSV WT, n=4; VSV hTim4, n=4). Equal volumes PBS were done as control (PBS WT,n=4; PBS hTim4, n=4). Ifng, Tnfα, Il2, Il12, Il5, Il6, Il9 and Il10 were examined in hTim4 and WT mice with or without VSV infection for 7days.The cytokine levels from scRNA-seq displayed by heatmap. (B) The cytokine levels of sera among various cell types. (C) The cytokine levels of sera between the DENV-2 and DMEM mice.

**Supporting Tables**

**Table S**1 The breading summary of hTim4 founder mice.

| F0 | DATE | SEX | F1  (hTim4+/all) | DATE | F2  (hTim4+ /all) | DATE | Comments |
| --- | --- | --- | --- | --- | --- | --- | --- |
| 17# | 15.12 | ♀ | 9/25 | 16.03~07 | 34/63 | 16.07~09 | ◇expression attenuation  ◇Sucking mice death |
| 31# | 16.01 | ♀ | - | 16.04~05 | - | - | No expression |
| 69# | 16.02 | ♀ | 24/37 | 16.05~12 | 43/94 | 17.03~07 | Stable expression |

**Table S2.** Primers used in this study.

| Target gene | primer | Sequence (5’-3’) |
| --- | --- | --- |
| hTim4 | Forward-1 | GCCCTGTCTGTACTCATCCTGG |
|  | Reverse-1 | GCGTGGTTGTTGAGGCTCTC |
|  | Forward-2 | TGCCCATCCTGGTCGAGCT |
|  | Reverse-2 | TTCACCAGGGTGTCGCCCTC |
| DENV-2 | Forward | CATGGGTAACTTATGGGAC |
|  | Reverse | GTTTCAGTTCGTGTCTCCA |
| IFN-β | Forward | CAGCTCCAAGAAAGGACGAAC |
|  | Reverse | GGCAGTGTAACTCTTCTGCAT |
| VSV | Forward | ACGGTTGGATGTGTCATGCT |
|  | Reverse | CTTTGAGGAGGGAAGCCTGG |
| Syk | Forward | CAGCCTACCTGAACGGAGTG |
|  | Reverse | GCAGAAAGCGTCGATGACAG |
| Il6 | Forward | TAGTCCTTCCTACCCCAATTTCC |
|  | Reverse | TTGGTCCTTAGCCACTCCTTC |
| Il10 | Forward | GCTCTTACTGACTGGCATGAG |
|  | Reverse | CGCAGCTCTAGGAGCATGTG |
| TBP | Forward | ACCCTTCACCAATGACTCCTATG |
|  | Reverse | ATGATGACTGCAGCAAATCG |

**Table S**3 The Pathological score and perfusion pressure in healthy, moderate and severe state.

| healthy | | moderate | | | severe | | |
| --- | --- | --- | --- | --- | --- | --- | --- |
| Grade | pressure | Grade | pressure | Drop rate | Grade | pressure | Drop rate |
| 0 | 141 | 2 | 111 | 19.04 | 3 | 70 | 48.94 |
| 0 | 134 | 2 | 105 | 23.41 | 3 | 98 | 28.52 |
| 0 | 132 | 2 | 96 | 29.98 | 3 | 80 | 41.65 |
| 0 | 136 | 2 | 101 | 26.33 | 3 | 81 | 40.92 |
| 0 | 145 | 2 | 97 | 29.25 | 3 | 67 | 51.13 |
| 0 | 142 | 2 | 96 | 29.98 |  |  |  |
| 0 | 139 | 2 | 97 | 29.25 |  |  |  |
| 0 | 138 | 2 | 96 | 29.98 |  |  |  |
| 0 | 130 | 2 | 99 | 27.79 |  |  |  |
| 0 | 128 | 2 | 96 | 29.98 |  |  |  |
| 0 | 143 | 2 | 96 | 29.98 |  |  |  |
| 0 | 135 | 2 | 97 | 29.25 |  |  |  |
| 0 | 138 | 2 | 96 | 29.98 |  |  |  |
| 0 | 140 | 2 | 97 | 29.25 |  |  |  |
| 0 | 145 | 2 | 98 | 28.52 |  |  |  |
| 0 | 137 | 2 | 97 | 29.25 |  |  |  |
| 0 | 134 | 2 | 96 | 29.98 |  |  |  |
| 0 | 130 | 2 | 96 | 29.98 |  |  |  |
| 0 | 134 | 2 | 96 | 29.98 |  |  |  |
| 0 | 136 |  |  |  |  |  |  |
| 0 | 141 |  |  |  |  |  |  |
| 0 | 138 |  |  |  |  |  |  |
| Total (Mean) | | | | | | | |
| 0 | 137.1 | 2 | 98.05 | 28.48 | 3 | 79.2 | 42.23 |

The criteria for pathological score was seen in Table S7. Lesion grade: healthy (0), mild lesion (1), moderate lesion (2), and severe lesion (3). The "perfusion pressure drop rate" referred to the percentage decrease in blood perfusion pressure observed in the moderate and severe groups relative to the healthy control group. The average pressure of healthy mice is 137.1 mmHg. The calculation formula was equal to [moderate/severe value – 137.1] ÷ 137.1. Regarding their correlation with clinical staging, "moderate" corresponds to DHF and "severe" corresponds to DSS. According to the surgical-specific criteria, a decrease of more than 20% (110 - 90 mmHg) from the preoperative baseline value is defined as hypotension (mild); < 90 mmHg is defined as severe hypotension mimicking DSS (severe), and the range between the two mimics DHF (moderate).

Table S4 The exact P values in this study.

| Figures | Statistical analyses | Groups venus | | P value |
| --- | --- | --- | --- | --- |
| Fig. 1D | unpaired Student's *t*-test | B: hTim4 *vs.* WT | | 0.0841 |
|  | unpaired Student's *t*-test | T: hTim4 *vs.* WT | | 0.0843 |
| Fig. 1H | unpaired Student's *t*-test | WT poly(I:C) *vs.* WT PBS | | p=0.2938 |
|  |  | hTim4 poly(I:C) *vs.* hTim4 PBS | | 0.7595 |
| Fig. 1I | unpaired Student's *t*-test | WT poly(I:C) *vs.* WT PBS | | 0.9375 |
|  |  | hTim4 poly(I:C) *vs.* hTim4 PBS | | 0.5470 |
| Fig. 1K | unpaired Student's *t*-test | WT poly(I:C) *vs.* WT PBS | | 0.3334 |
|  |  | hTim4 poly(I:C) *vs.* hTim4 PBS | | 0.5610 |
| Fig.1N | unpaired Student's *t*-test | 3 day WT VSV *vs.* WT PBS | | 0.2843 |
|  |  | 3 day hTim4 VSV *vs.* hTim4 PBS | | 0.7538 |
|  |  | 7 day WT VSV *vs.* WT PBS | | 0.0297 |
|  |  | 7 day hTim4 VSV *vs.* hTim4 PBS | | 0.4774 |
|  |  | 14 day WT VSV *vs.* WT PBS | | 0.3022 |
|  |  | 14 day hTim4 VSV *vs.* hTim4 PBS | | 0.7294 |
| Fig.1O |  | 3 day WT VSV *vs.* WT PBS | | 0.3482 |
|  |  | 3 day hTim4 VSV *vs.* hTim4 PBS | | 0.3172 |
|  |  | 7 day WT VSV *vs.* WT PBS | | <0.0001 |
|  |  | 7 day hTim4 VSV *vs.* hTim4 PBS | | 0.0173 |
|  |  | 14 day WT VSV *vs.* WT PBS | | 0.1288 |
|  |  | 14 day hTim4 VSV *vs.* hTim4 PBS | | 0.0787 |
| Fig.1P |  | 3 day WT VSV *vs.* WT PBS | | <0.0001 |
|  |  | 3 day hTim4 VSV *vs.* hTim4 PBS | | 0.0703 |
|  |  | 7 day WT VSV *vs.* WT PBS | | 0.1904 |
|  |  | 7 day hTim4 VSV *vs.* hTim4 PBS | | 0.6216 |
|  |  | 14 day WT VSV *vs.* WT PBS | | 0.0018 |
|  |  | 14 day hTim4 VSV *vs.* hTim4 PBS | | 0.0697 |
| Fig.1Q |  | 3 day WT VSV *vs.* WT PBS | | 0.0868 |
|  |  | 3 day hTim4 VSV *vs.* hTim4 PBS | | 0.5761 |
|  |  | 7 day WT VSV *vs.* WT PBS | | 0.2201 |
|  |  | 7 day hTim4 VSV *vs.* hTim4 PBS | | 0.5517 |
|  |  | 14 day WT VSV *vs.* WT PBS | | 0.1190 |
|  |  | 14 day hTim4 VSV *vs.* hTim4 PBS | | 0.0390 |
| Fig.1S | unpaired Student's *t*-test | PLT: hTim4 *vs.* WT | | 0.3937 |
|  |  | MPV: hTim4 *vs.* WT | | 0.0683 |
|  |  | PDW: hTim4 *vs.* WT | | 0.7430 |
|  |  | PCT: hTim4 *vs.* WT | | 0.9635 |
| Fig.1T | unpaired Student's *t*-test | APTT: hTim4 *vs.* WT | | 0.0568 |
| Fig.1U | unpaired Student's *t*-test | PT: hTim4 *vs.* WT | | 0.5407 |
| Fig.2B | unpaired Student's *t*-test | hTim4-Fc(0.2) *vs.* hTim4-Fc(0)  hTim4-Fc(1) *vs.* hTim4-Fc(0)  hTim4-Fc(5) *vs.* hTim4-Fc(0)  hTim4-Fc(1) *vs.* hTim4-Fc(0.2)  hTim4-Fc(5) *vs.* hTim4-Fc(1) | | 0.0062 |
|  |  |  |  | <0.0001 |
|  |  |  |  | 0.0122 |
|  |  |  |  | 0.2763 |
|  |  |  |  | 0.0477 |
| Fig.2D | unpaired Student's *t*-test | Spleen: hTim4 *vs.* WT | | <0.0001 |
|  |  | Liver: hTim4 *vs.* WT | | 0.0001 |
| Fig.2E | unpaired Student's *t*-test | Spleen: hTim4 *vs.* WT | | <0.0001 |
|  |  | Liver: hTim4 *vs.* WT | | 0.0055 |
| Fig.2F | Two way ANOVA | Spleen: hTim4 *vs.* WT | | P<0.0001 |
| Fig.2G | Two way ANOVA | Liver: hTim4 *vs.* WT | | P=0.0018 |
| Fig.2I | Two way ANOVA | 100 *vs.* 50 | | <0.0001 |
|  | Two way ANOVA | 500 *vs.* 50 | | <0.0001 |
|  | Two way ANOVA | 500 *vs.* 100 | | <0.0001 |
| Fig.2J | One way ANOVA | D6: 50 100 500 | | <0.0001 |
|  |  | D8: 50 100 500 | | <0.0001 |
|  |  | D10: 50 100 500 | | 0.0005 |
|  |  | D12: 50 100 500 | | 0.4461 |
|  |  | D14: 50 100 500 | | 0.6671 |
|  |  | D16: 50 100 500 | | 0.5026 |
| Fig.2K | Mantel-Cox | DENV-2 hTim4 *vs.* WT | | <0.0001 |
| Fig.2L | Two way ANOVA | WT PBS WT DENV-2 hTim4 PBS hTim4 DENV-2 | | <0.0001 |
| Fig.2M | Two way ANOVA | DENV-2 hTim4 *vs.* DENV-2 WT | | 0.0002 |
| Fig.2N | unpaired Student's *t*-test | 2 day: DENV-2 hTim4 *vs.* WT | | 0.0542 |
|  |  | 4 day: DENV-2 hTim4 *vs.* WT | | <0.0001 |
|  |  | 6 day: DENV-2 hTim4 *vs.* WT | | 0.0020 |
|  |  | 9 day: DENV-2 hTim4 *vs.* WT | | <0.0001 |
| Fig.3A | unpaired Student's *t*-test | hTim4 DENV-2 *vs.* DMEM | | <0.0001 |
| Fig.3C | Two way ANOVA | DENV-2 hTim4 *vs.* DENV-2 WT | | <0.0001 |
| Fig.3D | Two way ANOVA | DENV-2 hTim4 *vs.* DENV-2 WT | | 0.0338 |
| Fig.3E | Two way ANOVA | DENV-2 hTim4 *vs.* DENV-2 WT | | 0.0004 |
| Fig.4A | unpaired Student's *t*-test | hTim4 DENV-2 *vs.* DMEM | | <0.0001 |
| Fig.4C | unpaired Student's *t*-test | Succumbed *vs.* DMEM | | 0.0324 |
| Fig.4D | unpaired Student's *t*-test | Succumbed *vs.* DMEM | | <0.0001 |
| Fig.4E | unpaired Student's *t*-test | Succumbed *vs.* DMEM | | 0.0021 |
| Fig.4H | unpaired Student's *t*-test | Systole: Moderate *vs.*Healthy | | <0.0001 |
|  |  | Systole: Severve *vs.*Healthy | | <0.0001 |
|  |  | Diastole: Moderate *vs.*Healthy | | 0.0055 |
|  |  | Diastole: Severve *vs.*Healthy | | 0.0009 |
| Fig.4J | unpaired Student's *t*-test (welch) | Cross: DENV-2 hTim4 *vs.* WT | | 0.2740 |
|  |  | Longitidual:DENV-2 hTim4 *vs.* WT | | 0.4081 |
| Fig.4K | unpaired Student's *t*-test (welch) | Cross: DENV-2 hTim4 *vs.* WT | | 0.3986 |
|  |  | Longitidual:DENV-2 hTim4 *vs.* WT | | 0.0701 |
| Fig.7A | unpaired Student's *t*-test | Il-2 | hTim4 *vs.* WT | 0.1516 |
|  |  |  | hTim4 DENV-2 *vs.* DMEM | 0.0535 |
|  |  | Il-12P40 | hTim4 *vs.* WT | 0.0006 |
|  |  |  | hTim4 DENV-2 *vs.* DMEM | 0.1339 |
|  |  | Il-12P70 | hTim4 *vs.* WT | <0.0001 |
|  |  |  | hTim4 DENV-2 *vs.* DMEM | 0.5482 |
|  |  | Ifn-γ | hTim4 *vs.* WT | 0.0057 |
|  |  |  | hTim4 DENV-2 *vs.* DMEM | 0.2097 |
|  |  | Il-5 | hTim4 *vs.* WT | 0.0029 |
|  |  |  | hTim4 DENV-2 *vs.* DMEM | 0.0075 |
|  |  | Il-6 | hTim4 *vs.* WT | 0.0263 |
|  |  |  | hTim4 DENV-2 *vs.* DMEM | 0.0082 |
|  |  | Il-9 | hTim4 *vs.* WT | 0.0698 |
|  |  |  | hTim4 DENV-2 *vs.* DMEM | 0.3965 |
|  |  | Il-10 | hTim4 *vs.* WT | 0.6624 |
|  |  |  | hTim4 DENV-2 *vs.* DMEM | 0.0168 |
|  |  | Mcp-1 (Ccl2) | hTim4 *vs.* WT | <0.0001 |
|  |  |  | hTim4 DENV-2 *vs.* DMEM | 0.0869 |
|  |  | Mip-1a (Ccl3) | hTim4 *vs.* WT | 0.0002 |
|  |  |  | hTim4 DENV-2 *vs.* DMEM | 0.1134 |
|  |  | Mip-1β (Ccl4) | hTim4 *vs.* WT | <0.0001 |
|  |  |  | hTim4 DENV-2 *vs.* DMEM | 0.0138 |
|  |  | Rantes (Ccl5) | hTim4 *vs.* WT | <0.0001 |
|  |  |  | hTim4 DENV-2 *vs.* DMEM | 0.0015 |
| Fig.7B | unpaired Student's *t*-test | WT | DENV-2 *vs.* DMEM | 0.9028 |
|  |  | hTim4 | DENV-2 *vs.* DMEM | 0.0088 |
| Fig.7C | unpaired Student's *t*-test | WT | DENV-2 *vs.* DMEM | 0.4717 |
|  |  | hTim4 | DENV-2 *vs.* DMEM | 0.0223 |
| Fig.7D | unpaired Student's *t*-test | WT | DENV-2 *vs.* DMEM | 0.6259 |
|  |  | hTim4 | DENV-2 *vs.* DMEM | 0.4541 |
| Fig.7E | unpaired Student's *t*-test | WT | DENV-2 *vs.* DMEM | 0.0195 |
|  |  | hTim4 | DENV-2 *vs.* DMEM | 0.0008 |
| Fig.7F | unpaired Student's *t*-test | Il-2: DENV-2 *vs.* Healthy | | 0.2520 |
|  |  | Il-12: DENV-2 *vs.* Healthy | | 0.2738 |
|  |  | Ifn-γ: DENV-2 *vs.* Healthy | | 0.0984 |
|  |  | Il-5: DENV-2 *vs.* Healthy | | 0.0131 |
|  |  | Il-6: DENV-2 *vs.* Healthy | | 0.1148 |
|  |  | Il-9:DENV-2 *vs.* Healthy | | 0.0364 |
|  |  | Il-10: DENV-2 *vs.* Healthy | | 0.0468 |
|  |  | Mcp-1(Ccl2): DENV-2 *vs.* Healthy | | 0.0075 |
|  |  | Mip-1a (Ccl3): DENV-2 *vs.* Healthy | | 0.1802 |
|  |  | Mip-1β (Ccl4): DENV-2 *vs.* Healthy | | 0.0184 |
|  |  | Rantes (Ccl5): DENV-2 *vs.* Healthy | | 0.4129 |

**Table S5 The materials in this manuscript.**

| **REAGENT** | **SOURCE** | **IDENTIFIER** |
| --- | --- | --- |
| **Chemicals** | | |
| Zoletil®50 | Virbac | N/a |
| xylazine | Toronto research chemicals | X748000 |
| O.C.T component | SAKURA | 4583 |
| RNA iso-plus | Takara | 9109 |
| Evans blue dye | Sigma | E2129 |
| formamide | Sigma | F7503 |
| **Virus strain** | | |
| Dengue DENV-2 Tr.1751 | Capital Medicinal University | N/a |
| **Critical commercial Assays** | | |
| RT reagent Kit with gDNA Eraser | Takara | RR047A |
| TB Green® Premix Ex Taq™ II | Takara | RR820A |
| APTT test Kit | Solarbio | BC8080 |
| PTT test Kit | Solarbio | BC8081 |
| TIANprep Rapid Mini Plasmid Kit | Tiangen | DP105 |
| RNeasy Plus Universal Mini Kit | Qiagen | 73404 |
| TruSeq Stranded Total RNA | Illumina | 20020596 |
| MEGAshortscript™ T7 Transcription Kit | Ambion | AM1354 |
| mMESSAGE mMACHINE™ | Ambion | AM1344 |
| Poly(A) Tailing Kit | Ambion | AM1350 |
| MEGAclear™ | Ambion | AM1908 |
| Rapid Mini Plasmid Kit | Tiangen | DP105 |
| Mouse Cytokine 23-plex Assay | Bio-Rad | #M60009RDPD |
| Bio-Plex Pro Human Cytokine 27-plex Assay | Bio-Rad | #M500KCAF0Y |
| Visium Spatial Gene Expression Slide & Reagent kit | 10X Genomics | PN-1000184 |
| Hematology analysis lysis buffer | Mindary | 52LH100 |
| Hematology analysis diluent | Mindary | 52D020 |
| **Vector plasmid** | | |
| pUC 19 vector Plasmid | Addgene | #50005 |
| pET-32a+ vector Plasmid | Novagen | 69015 |
| **Antibody** | | |
| anti-Mouse CD45 antibody | Biolegend | 103132 |
| anti-Mouse CD19 antibody | Biolegend | 115520 |
| anti-Mouse CD49b antibody | Biolegend | 108920 |
| Anti-Albumin antibody | Sigma | SAB3500217 |
| Anti-Dengue Virus NS1 antibody | Abcam | ab41616 |
| Anti-CD31 antibody | Abcam | AB182981 |
| Anti-VE Cadherin antibody | Abcam | ab205336 |
| Anti-CD105 antibody | Abcam | ab221675 |
| **REAGENT** | **SOURCE** | **IDENTIFIER** |
| Anti-TIM 4 antibody | Abcam | ab222093 |
| Anti-Villin antibody | Abcam | ab97512 |
| Donkey Anti-Rabbit IgG H&L Alexa Fluor® 647 | Abcam | ab150075 |
| Goat Anti-Mouse IgG H&L (Alexa Fluor® 568) | Abcam | ab175473 |
| Goat Anti-Rat IgG H&L (Alexa Fluor® 594) | Abcam | ab150160 |
| Goat Anti-Chicken IgY H&L (Alexa Fluor® 555) | Abcam | ab150170 |
| TruSeq Stranded Total RNA Library Prep Gold | Illumina | 20020598 |
| HRP- labeled goat anti-rabbit IgG | Service Bio | GB23303 |

**Table S6 The criteria for “slow action", “limb paralysis” or "ataxia.**

| Grade | 0 | 1 | 2 | 3 | 4 | 5 |
| --- | --- | --- | --- | --- | --- | --- |
| slow action | normal | mildly impaired | sluggish | moderately impaired | severely impaired | immobile |
| limb paralysis | normal | mild weakness | moderate paralysis | severe paralysis | unilateral paralysis | valgus deformity |
| ataxia | normal | mildly unsteady | moderately unsteady | tremulous | staggering gait | choreiform movements |

**Table S7 The criteria for pathological score.**

| Pathological score (Grade) | Inflammation | Intestinal villus injury  Nuclear atrophy disordered arrangement  Cytoplasmic depression  Cell shedding. | Hemorrhage  Plasma leakage  Albumin exudation, etc | Total |
| --- | --- | --- | --- | --- |
| normal (0) | 0 | 0 | 0 | 0 |
| mild lesion (1) | 0~1 | 0~1 | 0~1 | 1~3 |
| moderate lesion (2) | 1~2 | 1~2 | 1~2 | 4~6 |
| Severe lesion (3). | 2~3 | 2~3 | 2~3 | 7~9 |
